# Pathological roles of the VEGF/SphK pathway in Niemann–Pick type C neurons

**DOI:** 10.1038/ncomms6514

**Published:** 2014-11-24

**Authors:** Hyun Lee, Jong Kil Lee, Min Hee Park, Yu Ri Hong, Hugo H. Marti, Hyongbum Kim, Yohei Okada, Makoto Otsu, Eul-Ju Seo, Jae-Hyung Park, Jae-Hoon Bae, Nozomu Okino, Xingxuan He, Edward H. Schuchman, Jae-sung Bae, Hee Kyung Jin

**Affiliations:** 1 Stem Cell Neuroplasticity Research Group, Kyungpook National University, Daegu 702-701, Korea; 2 Department of Laboratory Animal Medicine, Cell and Matrix Research Institute, College of Veterinary Medicine, Kyungpook National University, Daegu 702-701, Korea; 3 Department of Physiology, Cell and Matrix Research Institute, School of Medicine, Kyungpook National University, Daegu 700-842, Korea; 4 Department of Biomedical Science, BK21 Plus KNU Biomedical Convergence Program, Kyungpook National University, Daegu 700-842, Korea; 5 Institute of Physiology and Pathophysiology, University of Heidelberg, Heidelberg 69120, Germany; 6 Graduate School of Biomedical Science and Engineering/College of Medicine, Hanyang University, Seoul 133-791, Korea; 7 Department of Physiology, School of Medicine, Keio University, Tokyo 160-8582, Japan; 8 Division of Stem Cell Therapy, Center for Stem Cell Biology and Regenerative Medicine, Institute of Medical Science, University of Tokyo, Tokyo 108-8639, Japan; 9 Department of Laboratory Medicine, Asan Medical Center, University of Ulsan College of Medicine, Seoul 138-736, Korea; 10 Department of Physiology, School of Medicine, Keimyung University, Daegu 704-701, Korea; 11 Department of Bioscience and Biotechnology, Graduate School of Bioresource and Bioenvironmental Sciences, Kyushu University, Fukuoka 812-8581, Japan; 12 Department of Genetics and Genomic Sciences, Icahn School of Medicine at Mount Sinai, New York, New York 10029, USA

## Abstract

Sphingosine is a major storage compound in Niemann–Pick type C disease (NP–C), although the pathological role(s) of this accumulation have not been fully characterized. Here we found that sphingosine kinase (SphK) activity is reduced in NP–C patient fibroblasts and NP–C mouse Purkinje neurons (PNs) due to defective vascular endothelial growth factor (VEGF) levels. Sphingosine accumulation due to inactivation of VEGF/SphK pathway led to PNs loss via inhibition of autophagosome–lysosome fusion in NP–C mice. VEGF activates SphK by binding to VEGFR2, resulting in decreased sphingosine storage as well as improved PNs survival and clinical outcomes in NP–C cells and mice. We also show that induced pluripotent stem cell (iPSC)-derived human NP–C neurons are generated and the abnormalities caused by VEGF/SphK inactivity in these cells are corrected by replenishment of VEGF. Overall, these results reveal a pathogenic mechanism in NP–C neurons where defective SphK activity is due to impaired VEGF levels.

## Abstract (web summary)

Sphingosine is abnormally accumulated in Niemann–Pick type C disease (NP–C), but the causes of this accumulation have not been fully characterized. Here the authors show that sphingosine kinase activity is reduced in NP–C patient fibroblasts and NP–C mouse neurons due to defective vascular endothelial growth factor levels, suggesting therapeutic avenues.

## Graphical abstract

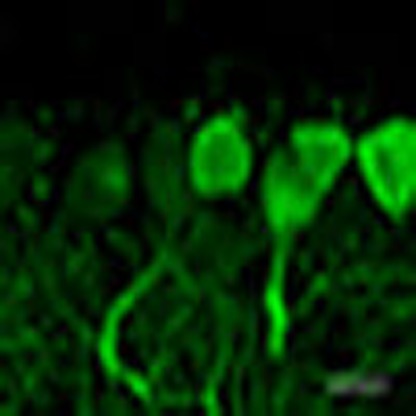

Niemann–Pick type C disease (NP–C) is an inherited lipid storage disorder that affects the central nervous system^1^,^2^,^3^. Recent studies have shown that sphingosine is a major and initiating storage compound in NP–C^3^,^4^. However, the underlying mechanism(s) leading to sphingosine storage, as well as its role in NP–C pathogenesis such as neuronal loss, remains largely unknown.

Our previous studies have shown that bone marrow mesenchymal stem cells (BM-MSCs) contribute to improved neurological function in the NP–C mice^5^,^6^. Furthermore, we have postulated that the prosurvival effects of BM-MSCs on NP–C Purkinje neurons (PNs) are paracrine effects that restore the sphingolipid imbalance, as evidenced by decreased sphingosine and increased sphingosine-1-phosphate (S1P) levels^7^. Therefore, we speculated that sphingolipid-modulating factors derived from BM-MSCs are potential therapeutic agents for this disease.

Sphingolipid-metabolizing enzymes control the cellular dynamic balance of bioactive lipids, including the proapoptotic compound sphingosine and the proliferative compound S1P^8^. Sphingosine kinase (SphK) is a key enzyme that converts sphingosine into S1P. SphK can be activated by numerous external stimuli^9^,^10^,^11^,^12^, resulting in a decrease in intracellular sphingosine and increase in S1P^13^.

On the basis of these concepts and findings, we hypothesized that defects of SphK activators could be involved in the pathogenesis of NP–C, and explored candidate therapeutic factors secreted by BM-MSCs that might influence the activation of SphK. Here we show that NPC1 deficiency markedly reduces vascular endothelial growth factor (VEGF) expression, and that decreased VEGF levels cause impaired SphK activity in PNs. Abnormal sphingosine storage by VEGF-mediated SphK inactivity causes a decreased PN survival via disruption of autophagosome–lysosome fusion. Further, replenishment of VEGF leads to restoration of SphK activity and improvement of pathology by binding to the VEGF receptor-2 (VEGFR2) in NP–C mice PNs as well as patient-specific cells, preventing sphingosine accumulation, autophagy dysfunction and abnormal calcium homeostasis.

## Results

### SphK activity is reduced in NP–C patients and NP–C mice

**Figure 1:**
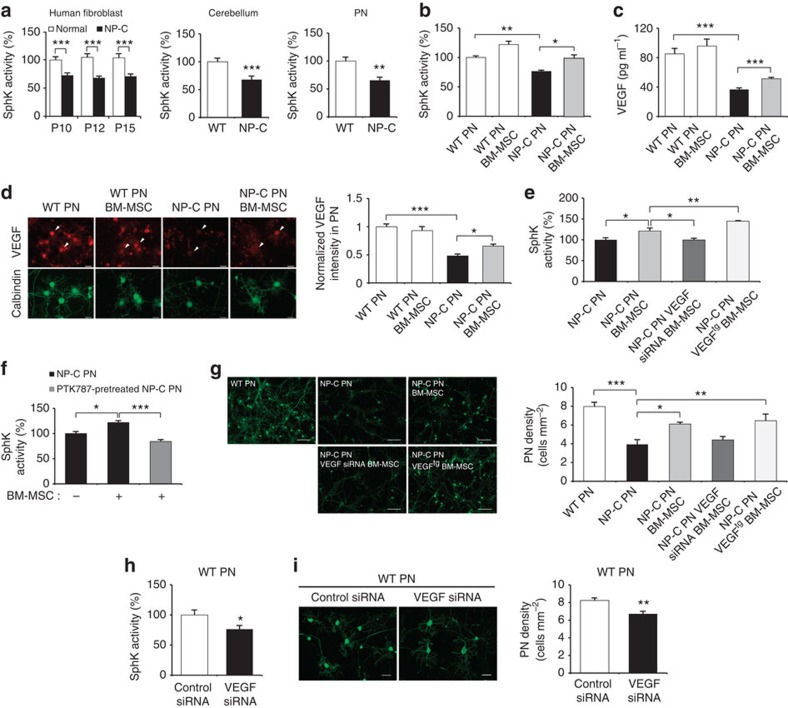
BM-MSC-derived VEGF restores SphK activity in NP–C mice PNs. (**a**) SphK activities between NP–C and control were analysed in human fibroblast (*n*=7 per group), mouse cerebellum tissue (*n*=7 per group) and primary mouse PN samples (*n*=9 per group). SphK activity did not show passage differences between NP–C and normal fibroblasts. (**b**) Three days after cocultures, we measured SphK activities in PNs derived from WT and NP–C mice (*n*=8 per group). (**c**) VEGF levels were measured in CM derived from PNs with or without BM-MSCs by ELISA (*n*=7 per group). (**d**) Primary cultures of NP–C PNs were immunostained with anti-calbindin and anti-VEGF (scale bar, 50 μm). Arrowheads indicate VEGF expression by PNs. Values represent normalized fluorescence intensities of VEGF in PNs (WT PN, *n*=8; and NP–C PN, *n*=9). (**e**) SphK activities were measured in NP–C PNs alone (*n*=7) and NP–C PNs cocultured with BM-MSCs, VEGF siRNA BM-MSCs and VEGF^tg^ BM-MSCs (*n*=8 per group). (**f**) Effect of the PTK787 on BM-MSCs mediated SphK activation. NP–C PNs were pretreated with PTK787 at 10 μM for 1 day and cocultured for 3 days with BM-MSCs, and then SphK activity was assayed (*n*=7 per group). (**g**) Representative images of PNs stained with anti-calbindin (scale bar, 100 μm). The mean number of PNs per squared millimetre was counted (*n*=8 per group). (**h**) Effect of VEGF knockdown on SphK activity in PNs (control, *n*=6; and VEGF siRNA, *n*=8 per group). (**i**) Representative images and quantification of neuronal survival in normal and VEGF-knockdown PNs (scale bar, 50 μm; *n*=8 per group). **a**,**h**,**i**, Student’s *t*-test. **b**–**g**, one-way analysis of variance, Tukey’s *post hoc* test. *P<0.05, **P<0.01, ***P<0.005. All error bars indicate s.e.m.

We first determined whether defects of SphK could be involved in NP–C and responsible for the elevated sphingosine. SphK was significantly decreased in fibroblasts from NP–C patients compared with normal control fibroblasts (Fig. 1a). These levels did not change as the passage numbers increased (Fig. 1a). SphK activity also was decreased in the cerebellum and primary cerebellar PNs from NP–C mice compared with those of wild-type (WT) mice (Fig. 1a). These results confirmed that SphK, a key enzyme in modulating the levels of sphingosine, is diminished in NP–C, and that the reduction of this activity may influence disease progression and/or pathogenesis.

### BM-MSC-derived VEGF restores SphK activity in NP–C mouse PNs

To examine whether bioactive, soluble factors released from BM-MSCs affected SphK activity in NP–C, we cocultured BM-MSCs with PNs using an indirect coculture system (see Methods). We found that when NP–C PNs were cocultured with BM-MSCs, their SphK activity was significantly increased (Fig. 1b). To identify the soluble factors that were released from the BM-MSCs and might be responsible for the increased SphK activity, we screened and compared the conditioned media (CM) of PNs grown with and without BM-MSCs using an antibody-based mouse cytokine array (Supplementary Fig. 1a,b). The CM of NP–C PNs cocultured with BM-MSCs revealed stronger signals in four array spots in comparison with the CM of NP–C PNs alone (Supplementary Fig. 1c,d). To confirm the secretion of these factors, we performed enzyme-linked immunosorbent assays (ELISA). Of the selected cytokines, only VEGF levels were significantly elevated in the CM of NP–C PNs cocultured with BM-MSCs. We also found that VEGF was significantly decreased in NP–C PNs cultured alone compared with WT PNs (Fig. 1c). To confirm these effects in PNs, we performed VEGF immunostaining. VEGF was normally expressed in PNs, but the expression levels were lower in NP–C PNs compared with WT PNs. When the NP–C PNs were cocultured with BM-MSCs, intensity of VEGF expression was increased (Fig. 1d). These data identified VEGF as a potential candidate molecule that could modulate SphK and may influence pathogenesis in NP–C PNs.

To further examine the effects of BM-MSC-derived VEGF on SphK activity in NP–C PNs, we used VEGF small interfering RNA (siRNA)-treated BM-MSCs and VEGF-overexpressing BM-MSCs (the latter derived from VEGF^tg^ mice; ref. 14; Supplementary Fig. 2a). As predicted, SphK activity was significantly increased in NP–C PNs cocultured with BM-MSCs and VEGF^tg^ BM-MSCs compared with NP–C PNs alone. However, the activity did not show any changes in NP–C PNs cocultured with VEGF siRNA-treated BM-MSCs (Fig. 1e). Consistent with this observation, sphingosine and S1P levels in the cocultured NP–C PNs were altered relative to the amount of VEGF released from the BM-MSCs (Supplementary Fig. 2b,c). We also performed S1P immunostaining in PNs. S1P was mainly expressed in PNs, and the expression was significantly increased in NP–C PNs cocultured with normal or VEGF^tg^ BM-MSCs. However, it was not increased when the cells were cocultured with VEGF siRNA-treated BM-MSCs (Supplementary Fig. 2d).

VEGF binds to two tyrosine kinase receptors, known as VEGFR1 and 2 (ref. 15). Among these receptors, VEGFR2 is highly expressed on PNs^16^. To examine whether VEGF from BM-MSCs improved the sphingolipid imbalance in NP–C PNs by binding to VEGFR2, we treated NP–C PNs with the VEGFR2 tyrosine kinase inhibitor PTK787 before coculturing^17^. We found that SphK activity and other sphingolipid metabolites in NP–C PNs were mediated by interactions of BM-MSC-derived VEGF and its receptor VEGFR2 (Fig. 1f; Supplementary Fig. 2e). These results indicated that BM-MSC-mediated restoration of abnormal SphK activity could be due the secreted VEGF binding to the VEGFR2 in NP–C PNs.

Next, to determine whether the VEGF-mediated SphK modulation by BM-MSCs promoted the survival of NP–C PNs, we determined cell counts after coculture. When NP–C PNs were cocultured with BM-MSCs or VEGF^tg^ BM-MSCs, the number of PNs was significantly increased. This effect was lower when VEGF siRNA BM-MSCs were cocultured with the NP–C PNs, although this did not reach statistical significance (Fig. 1g).

Finally, to gain more direct insights into the relationship between VEGF and SphK activity in NP–C PNs, we treated WT PNs with VEGF siRNA and determined the changes of sphingolipid factors. VEGF siRNA treatment of WT PNs strongly reduced SphK levels and led to elevation of sphingosine and reduction of S1P, similar to NP–C PNs (Fig. 1h; Supplementary Fig. 2f,g). The survival of PNs was also significantly decreased following VEGF siRNA transfection (Fig. 1i). These results suggested that inactivation of VEGF may lead to reduced SphK activity in NP–C PNs.

### VEGF from BM-MSCs reduces pathology in PNs of NP–C mice

**Figure 2:**
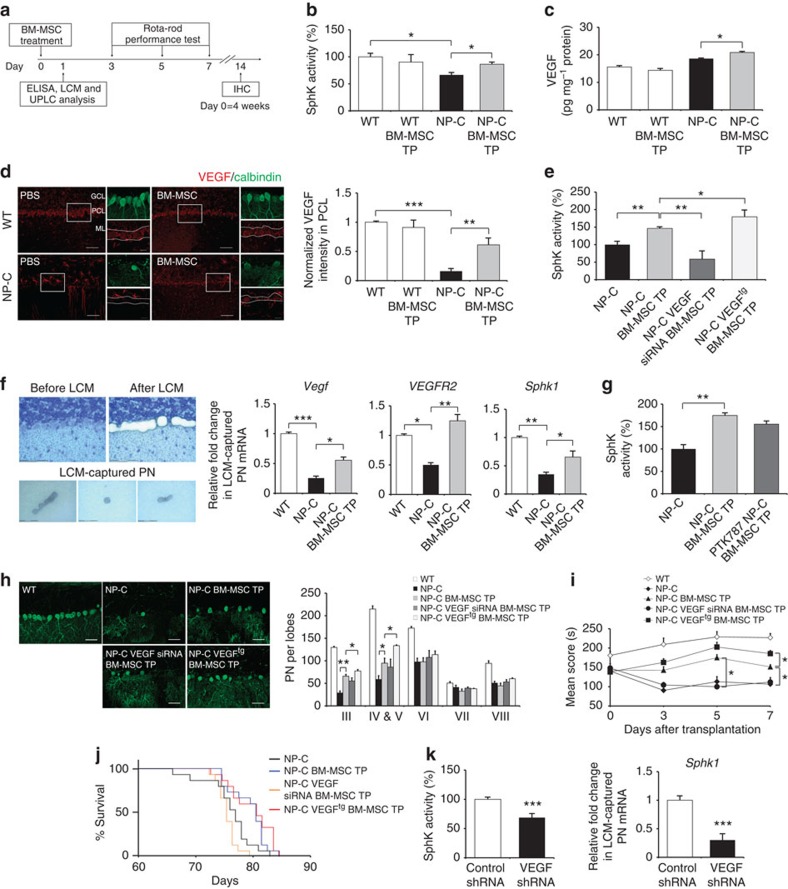
VEGF from BM-MSCs reduces pathology in PNs of NP–C mice. (**a**) Protocol of BM-MSC treatment in NP–C mice. (**b**,**c**) SphK activity (*n*=7 per group; **b**) and VEGF levels (*n*=8 per group; **c**) were estimated in the cerebellums of WT and NP–C mice after BM-MSC treatment. (**d**) Cerebellar sections were stained with anti-calbindin and anti-VEGF (low-magnification scale bar, 50 μm; high-magnification scale bar, 20 μm). Values represent normalized VEGF fluorescence intensities in PCL (*n*=7 per group). (**e**) SphK activities were measured in the cerebellums of NP–C mice treated with PBS (*n*=6), BM-MSCs, VEGF siRNA BM-MSCs and VEGF^tg^ BM-MSCs (*n*=8 per group). (**f**) Left, isolation of mouse PNs using LCM (scale bar, 75 μm). Right, mRNA level of *Vegf*, *VEGFR2* and *Sphk1* on LCM-captured PNs samples (*n*=7 per group). (**g**) NP–C mice were treated daily with the PTK787 at 100 mg kg^−1^ or PBS, starting 2 days before the BM-MSC transplantation. One day after BM-MSC treatment, SphK activity was estimated (NP–C, *n*=7; NP–C BM-MSC TP, *n*=8 per group). (**h**) Cerebellar sections were stained with anti-calbindin (scale bar, 50 μm), and the number of calbindin-positive PNs were quantified (*n*=7 per group). (**i**) Rota-rod scores of mice were averaged and plotted beginning 3 days after transplantation (*n*=15 per group). (**j**) Survival curve of NP–C mice (*n*=15 per group). Treatment with BM-MSCs and VEGF^tg^ BM-MSCs resulted in significantly increased survival compared with PBS treatment (*P*=0.0194 and *P*=0.0055, respectively; log-rank test). (**k**) Effect of VEGF knockdown on SphK activity. Left, after intracerebellar injection of control (*n*=7) or VEGF shRNA (*n*=8) in mice, SphK activities were measured in the cerebellums. Right, relative levels of *Sphk1* mRNA from LCM-captured PNs samples (*n*=7 per group). **b**–**i**, one-way analysis of variance, Tukey’s *post hoc* test. **k**, Student’s *t*-test. *P<0.05, **P<0.01, ***P<0.005. All error bars indicate s.e.m.

To examine the *in vivo* effects of VEGF derived from BM-MSCs on SphK activity of PNs, we transplanted BM-MSCs into the cerebellum of NP–C mice (Fig. 2a). At one day after BM-MSC transplantation, SphK activity was significantly increased in the cerebellum of NP–C mice compared with phosphate-buffered saline (PBS)-infused counterparts (Fig. 2b). BM-MSC transplantation also increased VEGF protein levels in the cerebellum of NP–C mice (Fig. 2c). The elevated expression of VEGF was significant in the Purkinje cell layer (PCL) of the NP–C mouse cerebellums, consistent with the decreased VEGF levels in non-treated NP–C PNs compared with WT (Fig. 2d). However, BM-MSCs did not increase SphK or VEGF levels in normal cerebellums, consistent with previous reports^6^,^18^.

We also transplanted VEGF siRNA BM-MSCs and VEGF^tg^ BM-MSCs into the cerebellum of NP–C mice. As predicted, at one day after transplantation, SphK activity was significantly increased in the cerebellum of NP–C mice treated with VEGF^tg^ BM-MSCs. However, mice treated with VEGF siRNA BM-MSCs showed significantly lower SphK activity (Fig. 2e). The sphingosine and S1P metabolites were also changed in NP–C PNs in relation to SphK and VEGF levels (Supplementary Fig. 3a,b). Similar effects were observed when S1P immunostaining was performed on the PN layer of NP–C mice following transplantation with VEGF siRNA or VEGF-overexpressing BM-MSCs (Supplementary Fig. 3c). To further confirm these effects, we used laser capture microdissection (LCM) to selectively isolate PNs (Supplementary Fig. 3d). We observed that expressions of *Vegf*, *VEGFR2* and *Sphk1* mRNAs were decreased in LCM-captured PNs from NP–C mice compared with that of WT mice. BM-MSC transplantation enhanced these expression levels in NP–C PNs (Fig. 2f). We also ascertained whether VEGFR2 was required for the activation of SphK in NP–C mice. As shown in Fig. 2g, SphK activity was significantly increased in the NP–C mice following BM-MSC treatment, whereas this effect was lower in NP–C mice treated with PTK787 before injecting BM-MSCs, although this did not reach statistical significance. S1P levels were moderately decreased with PTK787 treatment, but sphingosine did not vary between the groups (Supplementary Fig. 3e).

Next, we evaluated the effects of VEGF on the NP–C phenotype in mice. Transplantation of VEGF^tg^ BM-MSCs improved NP–C pathology as measured by increased number of calbindin-positive PNs on 14 days after treatment (Fig. 2h), and also enhanced the Rota-rod performance (Fig. 2i). These effects were less in the VEGF siRNA BM-MSC-treated group. Rota-rod performance also diminished in the VEGF siRNA BM-MSC-treated NP–C mice over time. Moreover, the lifespan of mice that had BM-MSC or VEGF^tg^ BM-MSC transplants was extended (Fig. 2j).

Finally, to determine whether the reduced VEGF levels in the cerebellums affected SphK activity, we injected VEGF short hairpin RNA (shRNA) into the cerebellum of WT mice and determined the changes of sphingolipid factors. Treatment with VEGF shRNA markedly reduced SphK activity and *Sphk1* mRNA levels (Fig. 2k; Supplementary Fig. 3f,g) and led to elevation of sphingosine and reduction of S1P (Supplementary Fig. 3h). These results suggested that inactivation of VEGF may lead to reduced SphK activity in NP–C mice, consistent with *in vitro* results. Together, these findings show a direct correlation between VEGF and SphK activity in PNs and suggest that abnormal sphingosine accumulation in NP–C may be due to the dysfunction of SphK activity by inactivated VEGF expression.

### NPC1 deficiency impairs VEGF/SphK activation in PNs

**Figure 3:**
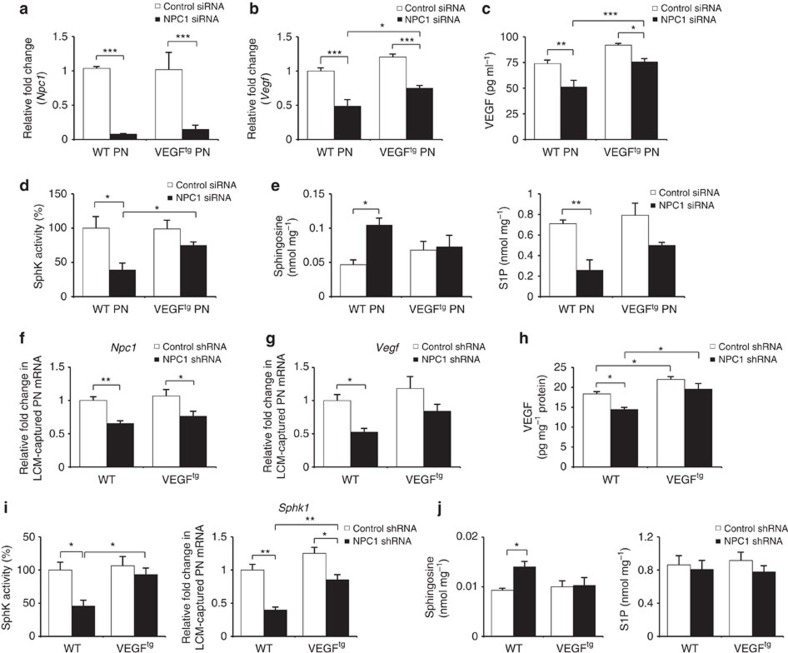
NPC1 knockdown reduces VEGF expression and SphK activity. (**a**–**c**) Primary cultures of normal and VEGF^tg^ PNs were transfected with control or NPC1 siRNA. Three days after transfection, we measured the levels of *Npc1* (**a**) and *Vegf* (**b**) mRNA and secreted VEGF protein (**c**) in PNs (*n*=7 per group). (**d**,**e**) SphK activity (**d**), sphingosine and S1P (**e**) were estimated in PNs transfected with control (*n*=6) or NPC1 siRNA (*n*=7). (**f**–**j**) Four-week-old WT and VEGF^tg^ mice were injected with control or NPC1 shRNA into the cerebellum. Mice were sacrificed at 3 days after the injection. *Npc1* (**f**) and *Vegf* (**g**) mRNA levels were estimated in LCM-captured PNs and VEGF protein levels (**h**) were measured in the cerebellums (*n*=7 per group). (**i**) Left, SphK activities were measured in the cerebellums (*n*=7 per group). Right, relative levels of *Sphk1* mRNA from LCM-captured PNs samples (*n*=8 per group). (**j**) Sphingosine and S1P were measured in the cerebellums (*n*=7 per group). **a**–**j**, one-way analysis of variance, Tukey’s *post hoc* test. *P<0.05, **P<0.01, ***P<0.005. All error bars indicate s.e.m.

We subsequently investigated the relationship between NPC1 and VEGF expression. NPC1 knockdown by siRNA markedly decreased VEGF expression in normal PNs. When NPC1 was knocked down in VEGF^tg^ PNs (derived from VEGF^tg^ mice), the decreased level of VEGF was lower than that of normal PNs (Fig. 3a–c). Moreover, NPC1 deficiency markedly inactivated SphK and led to sphingolipid imbalance. In VEGF^tg^ PNs, however, moderate changes were observed (Fig. 3d,e). We next tested whether NPC1 deficiency affected VEGF expression in the cerebellums of WT mice using NPC1 shRNA. Intracerebellar injection of NPC1 shRNA, which decreased *Npc1* mRNA expression in the LCM-captured PNs (Fig. 3f), reduced VEGF expression (Fig. 3g,h). Consistently, NPC1 deficiency significantly decreased SphK activity and *Sphk1* mRNA expression and led to elevation of sphingosine and reduction of S1P in the cerebellums (Fig. 3i,j). These effects were moderated in VEGF^tg^ mice (Fig. 3g–j). Overall, these results indicated that knockdown of NPC1 may lead to reduced VEGF expression, and these reductions subsequently decreased SphK activity in PNs.

### VEGF overexpression ameliorates NP–C pathology in mice

**Figure 4:**
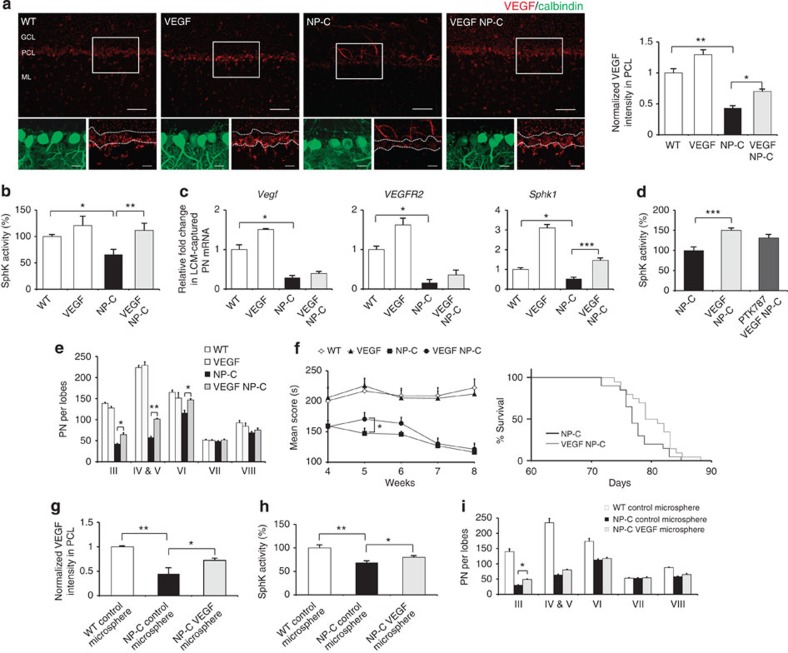
Replenishment of VEGF ameliorates NP–C pathology in mice. (**a**) Cerebellar sections from 6-week-old WT, VEGF, NP–C and VEGF/NP–C mice were immunostained with anti-calbindin and anti-VEGF (low-magnification scale bar, 50 μm; high-magnification scale bar, 20 μm). The average VEGF fluorescence intensity within the PCL was measured (WT, *n*=7; VEGF, *n*=7; NP–C, *n*=9; and VEGF/NP–C, *n*=9). (**b**) SphK activities were measured in cerebellums derived from 6-week-old WT, VEGF, NP–C and VEGF/NP–C mice (*n*=8 per group). (**c**) Quantitative real-time PCR for *Vegf*, *VEGFR2* and *Sphk1* mRNA in LCM-captured PNs in 6-week-old WT, VEGF, NP–C and VEGF/NP–C mice (WT, *n*=6; VEGF, *n*=6; NP–C, *n*=8; and VEGF/NP–C, *n*=8). (**d**) VEGF/NP–C mice were treated daily with the PTK787 at 100 mg kg^−1^ or PBS vehicle control for 3 days before sacrifice (6-week-old), and SphK activity was estimated in cerebellums (*n*=7 per group). (**e**) Cerebellar sections were immunostained with anti-calbindin and the number of calbindin-positive PNs was quantified (WT, *n*=7; VEGF, *n*=7; NP–C, *n*=8; and VEGF/NP–C, *n*=8). (**f**) Left, beginning at 4 weeks of age, Rota-rod scores were averaged and plotted (*n*=15 per group). Right, survival curves of NP–C and VEGF/NP–C mice (*P*=0.0548; log-rank test, *n*=15 per group). (**g**) Cerebellar sections from WT and NP–C mice transplanted with VEGF-loaded or control microspheres were stained with anti-calbindin and anti-VEGF. The average VEGF fluorescence intensity within the PCL was measured (*n*=7 per group). (**h**) SphK activity was estimated in the cerebellums of WT and NP–C mice at one day after treatment. (**i**) Cerebellar sections were prepared at 2 weeks after transplantation and immunostained with anti-calbindin. The calbindin-positive PNs were counted (*n*=7 per group). **a**–**i**, one-way analysis of variance, Tukey’s *post hoc* test. *P<0.05, **P<0.01, ***P<0.005. All error bars indicate s.e.m.

The VEGF-mediated SphK reduction in NP–C PNs prompted us to examine possible genetic implications of this pathway. To increase VEGF in NP–C mice, we generated *VEGF*^*tg*^/*Npc1*^*−/−*^ mice (Supplementary Fig. 4a). VEGF is widely expressed in neurons, glia and endothelial cells^19^,^20^, with strong expression in PNs. In NP–C cerebellum, however, VEGF was mainly expressed in the granular layer and significantly decreased in the PCL. VEGF/NP–C mice showed increased expression of VEGF in the PCL compared with NP–C mice (Fig. 4a).

To examine whether genetically increasing VEGF affects SphK activity in NP–C PNs, we analysed cerebellum samples derived from 6-week-old WT, VEGF, NP–C and VEGF/NP–C mice. Compared with NP–C mice, VEGF/NP–C mice showed significantly increased SphK activity and decreased sphingosine accumulation (Fig. 4b; Supplementary Fig. 4b). Cerebellar S1P levels did not vary between the NP–C and VEGF/NP–C mice, although S1P levels in the PCL was increased in VEGF/NP–C mice (Supplementary Fig. 4b,d). Sphingomyelin and unesterified cholesterol levels were also significantly decreased in VEGF/NP–C mice, but glycosphingolipid (GSL) levels did not vary between the groups (Supplementary Fig. 4c,e,f). These results revealed that genetic VEGF overexpression could reverse the SphK abnormality and abnormal lipid accumulation in NP–C. To confirm VEGF-mediated SphK activation within PNs, we measured the *Vegf*, *VEGFR2* and *Sphk1* mRNA levels in LCM-captured PNs from these mice. LCM-captured PNs from VEGF/NP–C showed slightly increased *Vegf* and *VEGFR2* mRNA levels and significantly enhanced *Sphk1* mRNA levels (Fig. 4c).

Next, to further investigate the subcellular distribution pattern of SphK activity, sphingosine and S1P, we isolated cytosolic-enriched and lysosome-enriched fractions from the cerebellums. SphK activity was increased in VEGF/NP–C-derived lysosomes and cytosol compared with NP–C-derived ones, although the degree of SphK increase was greater in the cytosol than lysosome. Accumulated sphingosine in NP–C was found in the lysosome. Lysosomal sphingosine levels were significantly decreased in the VEGF/NP–C, whereas S1P levels did not vary between the groups (Supplementary Fig. 4g). Taken together, these results suggested that VEGF leads to activated SphK in the lysosome and cytosol and that activated SphK decreased lysosomal sphingosine accumulation in NP–C. We next observed whether the activation of VEGFR2 was required for the activation of SphK in VEGF/NP–C mice. Increased SphK activity was lower in VEGF/NP–C mice treated with the PTK787, although this did not reach statistical significance (Fig. 4d). Sphingosine levels also were moderately increased, but S1P levels did not vary between the groups (Supplementary Fig. 4h). PN survival was significantly improved in the VEGF/NP–C mice (Fig. 4e), and there were improvements in the Rota-rod score of 5-week-old VEGF/NP–C mice compared with NP–C mice (Fig. 4f, left). The lifespan of the VEGF/NP–C mice was slightly increased (Fig. 4f, right). We also found that BM-MSC transplantation is more effective in SphK modulation than genetic replenishment of VEGF (see Fig. 2). These results suggested that other factors secreted by BM-MSCs might also contribute to SphK activation.

We next tested whether pharmacologic delivery of recombinant VEGF is beneficial to NP–C pathology. Since the injected recombinant VEGF exerted a short-lived effect^21^, to overcome this obstacle we generated a microsphere system that allows localized and sustained VEGF release (Supplementary Fig. 5a). We injected 3 mg of VEGF-loaded microspheres or control microspheres into the cerebellum of 4-week-old NP–C and WT mice. Two weeks after treatment, NP–C mice transplanted with VEGF-loaded microspheres had higher levels of VEGF expression in the PCLs (Fig. 4g), exhibited increased SphK activity (Fig. 4h) and decreased sphingosine levels (Supplementary Fig. 5b) in their cerebellums. S1P levels in cerebellum and expression in PNs were also increased by VEGF-loaded microsphere treatment (Supplementary Fig. 5b,c). Further, the VEGF-loaded microsphere-treated NP–C mice showed significantly improved PN survival (Fig. 4i).

### VEGF overexpression reverses defective autophagy in NP–C mice

**Figure 5:**
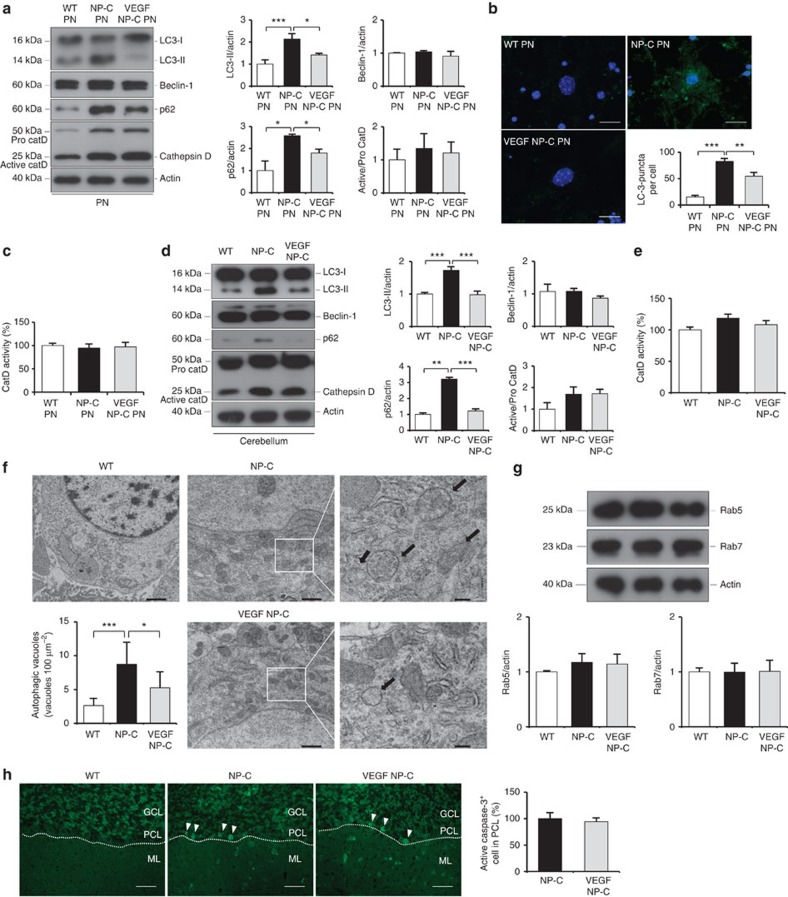
VEGF replenishment reverses defective autophagy in NP–C mice. (**a**) Western blot analysis of LC3, beclin-1, p62 and cathepsin D in primary cultured PNs derived from WT, NP–C and VEGF/NP–C mice (WT, *n*=5; NP–C, *n*=6; and VEGF/NP–C, *n*=6). (**b**) Immunocytochemistry of LC3 in WT, NP–C and VEGF/NP–C PNs (*n*=6 per group; scale bar, 20 μm). (**c**) Cathepsin D activity in primary cultured PNs (WT, *n*=5; NP–C, *n*=6; and VEGF/NP–C, *n*=6). (**d**) Western blot analysis of LC3, beclin-1, p62 and cathepsin D in the cerebellums of 6-week-old WT, NP–C and VEGF/NP–C mice (WT, *n*=6; NP–C, *n*=7; and VEGF/NP–C, *n*=7). (**e**) Cathepsin D activity in the cerebellums of WT, NP–C and VEGF/NP–C mice (WT, *n*=5; NP–C, *n*=6; and VEGF/NP–C, *n*=6). (**f**) EM images and quantification data of the cerebellum (*n*=5 per group; low-magnification scale bar, 1 μm; high-magnification scale bar, 200 nm). Arrow indicates autophagic vacuole. (**g**) Western blot analysis of Rab5 and Rab7 levels in the cerebellum (*n*=6 per group). (**h**) Cerebellar sections were immunostained with anti-active caspase-3 and the number of active caspase-3-positive cells in PCL was quantified (*n*=5 per group; scale bar, 50 μm). **a**–**g**, one-way analysis of variance, Tukey’s *post hoc* test. **h**, Student’s *t*-test. *P<0.05, **P<0.01, ***P<0.005. All error bars indicate s.e.m.

Autophagy, a major degradative pathway of the lysosomal system, is known to be markedly impaired in NP–C. These defects lead to loss of PNs in NP–C^22^. To examine whether increased PN survival in VEGF/NP–C mice was related to autophagy, we first measured LC3-II levels. Consistent with previous result^22^, we found that the LC3-II levels were significantly increased in PNs and cerebellum samples derived from NP–C mice. This enhanced LC3-II level was reduced in VEGF/NP–C mice (Fig. 5a,b,d). The level of beclin-1 did not vary between the groups (Fig. 5a,d). The levels of cathepsin D, a lysosomal hydrolase, were slightly increased in NP–C mice compared with WT mice (Fig. 5a,d). However, the activity of cathepsin D was not changed between the groups (Fig. 5c,e). This result indicated that the elevated levels of cathepsin D in NP–C mice did not ultimately translate into a significant increase in enzyme activity. Cathepsin D levels in VEGF/NP–C mice were comparable to that of NP–C mice, indicating that increased VEGF in NP–C mice did not influence the cathepsin D expression (Fig. 5a,c–e). The level of p62 was significantly higher in NP–C mice compared with WT mice, but was decreased in VEGF/NP–C mice (Fig. 5a,d). We also performed transmission electron microscopic (EM) analysis using mouse cerebellum samples to corroborate the immunoblotting results. NP–C mice brains showed massive increases of autophagic vacuoles, while brains of VEGF/NP–C mice represented a reduced number of these vesicles (Fig. 5f).

Next, to determine whether the endocytic pathway was affected by VEGF overexpression in NP–C mice, we examined Rab5 and Rab7 expression in our animals. The levels of these proteins showed no differences between the groups (Fig. 5g). Apoptotic cells, as judged by active caspase-3, did not show any differences between NP–C and VEGF/NP–C mice (Fig. 5h). Our results showed that endocytic pathway and apoptosis were not the main mechanisms of increased PN survival in VEGF/NP–C mice.

### Impaired VEGF/SphK pathway causes defective autophagic flux

**Figure 6:**
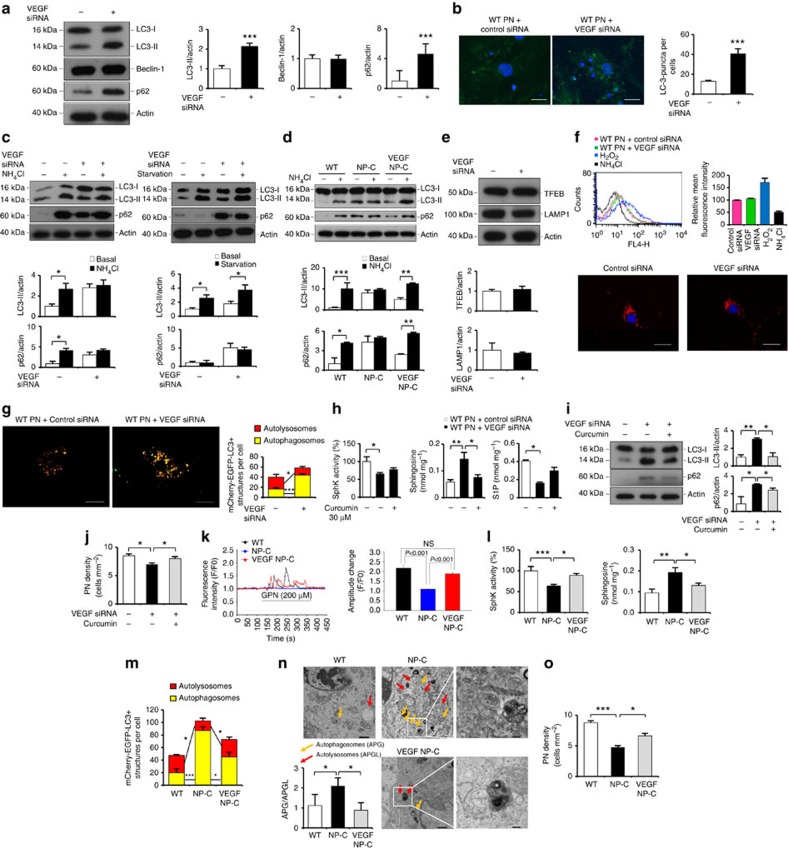
VEGF/SphK inactivity impairs autophagic flux. (**a**) Western blots of LC3, beclin-1 and p62 in PNs after VEGF knockdown (*n*=6 per group). (**b**) Immunocytochemistry of LC3 in PNs after VEGF knockdown (*n*=6 per group; scale bar, 20 μm). (**c**) Autophagic flux assay. Western blots of LC3 and p62 in PNs (*n*=6 per group). (**d**) Western blots of LC3 and p62 in cultured PNs in the presence of NH_4_Cl (*n*=5 per group). (**e**) Western blots of TFEB and Lamp1 in VEGF-knockdown PNs (control, *n*=5 and VEGF siRNA, *n*=6). (**f**) Effect of VEGF knockdown on lysosomal pH. PNs stained with LysoTracker red (*n*=5 per group; scale bar, 20 μm). (**g**) Fluorescence analysis of autophagosomes and autolysosomes (control, *n*=7 and VEGF siRNA, *n*=8; scale bar, 10 μm). (**h**) Sphk activity, sphingosine and S1P levels in PNs after VEGF knockdown in the presence of curcumin (*n*=8 per group). (**i**) Western blot analysis of LC3 and p62 in VEGF-knockdown PNs treated with curcumin (control, *n*=7; VEGF siRNA, *n*=8; and VEGF siRNA/curcumin, *n*=8). (**j**) Survival of VEGF-knockdown PNs treated with curcumin (*n*=8 per group). (**k**) Left, representative traces showing intracellular [Ca^2+^] changes monitored in single fluo-4-loaded PNs. Right, maximal peak fluorescence changes were determined as the differences between basal and the maximum fluorescence (*n*=10 cells per group). (**l**) Sphk activity and sphingosine levels were measured in cultured PNs (*n*=8 per group). (**m**) Quantification of autophagosomes and autolysosomes in primary cultured PNs (WT, *n*=7; NP–C, *n*=7; and VEGF/NP–C, *n*=8). (**n**) EM analysis of the PNs (*n*=5 per group; low-magnification scale bar, 1 μm; high-magnification scale bar, 200 nm). (**o**) Survival of primary cultured PNs (WT, *n*=6; NP–C, *n*=8; and VEGF/NP–C, *n*=8). **a**,**b**,**e**,**g**, Student’s *t*-test. **c**,**d**,**f**,**h**–**o**, one-way analysis of variance, Tukey’s *post hoc* test. *P<0.05, **P<0.01, ***P<0.005. All error bars indicate s.e.m.

Improved autophagic degradation in the VEGF/NP–C mice prompted us to analyze whether VEGF-mediated sphingolipid changes affect autophagy activity. First, to unravel the mechanistic link between VEGF levels and autophagic dysfunction, VEGF was depleted in the WT PNs by siRNA treatment. Knockdown of VEGF caused increased accumulation of LC3-II and p62 (Fig. 6a,b). Beclin-1 expression was not affected by VEGF knockdown (Fig. 6a), indicating that the accumulation of autophagosomes was not due to the biogenesis pathway.

The accumulation of autophagosomes can occur due to either an increase in their rate of formation or a reduction in their rate of degradation^23^. To distinguish between these two events, we examined the effects of VEGF knockdown on LC3-II levels in WT PNs in the presence or absence of NH_4_Cl that blocks autophagic degradation but does not affect autophagosome formation. VEGF knockdown increased accumulation of LC3-II. This level was not further increased by NH_4_Cl treatment (Fig. 6c, left). In contrast, VEGF depletion in serum starvation culture resulted in a significant increase in LC3-II levels (Fig. 6c, right). These observations were also supported by levels of p62 (Fig. 6c). These results suggested that VEGF depletion influences at a late step of autophagy. We also performed autophagy flux assay in WT, NP–C and VEGF/NP–C mice PNs. Under basal condition, NP–C PNs showed significantly increased LC3-II and p62 levels compared with WT PNs. NH_4_Cl-induced lysosome inhibition led to marked increase of LC3-II and p62 levels in the WT PNs, but this increase was significantly less in the NP–C PNs (Fig. 6d). VEGF/NP–C PNs showed similar pattern in LC3-II and p62 increase compared with WT cells (Fig. 6d). Taken together, these results indicated that reduced VEGF levels in NP–C PNs caused a defect of autophagic degradation, but not induction.

Our findings that depletion of VEGF affects autophagic degradation prompted us to more closely examine how VEGF might influence in defective autophagic degradation. We first examined the transcription factor EB (TFEB), which coordinates lysosomal formation^24^. VEGF depletion in WT PNs did not affect the levels of TFEB and Lamp1, indicating that VEGF did not impair lysosome biogenesis (Fig. 6e). Next, we assessed alteration in lysosomal pH using the acidotropic dye LysoTracker red. H_2_O_2_- and NH_4_Cl-treated cells were used as positive and negative controls, respectively. VEGF siRNA-treated PNs exhibited a similar fluorescence to control siRNA-treated PNs, indicating that VEGF did not affect lysosomal acidification (Fig. 6f).

Following the initiation of the phagophores, autophagosomes undergo a stepwise maturation process from early to late autophagosomes, which ultimately fuse with lysosomes to form autolysosomes. To study the effect of VEGF depletion on the maturation of autophagosomes, we used mCherry-EGFP-LC3 reporter^25^. Before fusion with lysosomes, the LC3-II-positive autophagosomes are shown by both GFP and mCherry signals as yellow puncta, and after fusion, autolysosomes are shown by only mCherry signals as red-only puncta because GFP loses its fluorescence in acidic pH. Compared with control PNs, VEGF siRNA-treated cells showed significantly increased yellow puncta (autophagosomes) and decreased mCherry-only puncta (autolysosomes), indicating that VEGF depletion inhibited the autophagosome–lysosome fusion (Fig. 6g).

Sphingosine accumulation can induce defective calcium release from the acidic compartment such as lysosome, which inhibits fusion of lysosome with other organelles^4^. As shown in Supplementary Figs 2g and 3h, decreased VEGF levels caused sphingosine accumulation in WT PNs. Thus, we hypothesized that defective lysosomal calcium release by VEGF-mediated sphingosine accumulation disturbs autophagosome–lysosome fusion and evokes the abnormal autophagosomes’ amassment. To test this hypothesis, we used the weaker sarcoplasmic reticulum ATPase antagonist curcumin, a natural product derived from turmeric^26^, which correct sphingolipid imbalance by increasing the cytosolic calcium release^4^. Importantly, abnormal sphingolipid levels in the VEGF siRNA-treated PNs were normalized after curcumin treatment (Fig. 6h). We also observed significantly decreased protein level of abnormal autophagic markers (Fig. 6i) and increased neuronal survival (Fig. 6j) in the VEGF-knockdown PNs after curcumin treatment compared with PNs with VEGF knockdown alone. Similar results were observed in the VEGF shRNA-treated mice after curcumin injection (Supplementary Fig. 6). To further examine these effects, we analysed calcium homeostasis in the primary cultured PNs derived from WT, NP–C and VEGF/NP–C mice. To specifically assess the lysosomal calcium content, we used Gly–Phe β-naphthylamide (GPN), which osmotically lyses cathepsin-containing lysosome^4^. We observed a reduction in NP–C PNs’ calcium release from lysosome compared with WT PNs, consistent with our previous study^7^. Notably, this reduction was corrected in VEGF/NP–C PNs (Fig. 6k). As expected, abnormal sphingosine accumulation was reduced in VEGF/NP–C cells by restoration of SphK activity (Fig. 6l). Moreover, VEGF/NP–C PNs showed decreased autophagosome (yellow LC3 puncta) accumulation (Fig. 6m). Decreased autophagosomes in the VEGF/NP–C PNs were further confirmed by EM analysis (Fig. 6n). The survival of PNs was also significantly increased in the VEGF/NP–C (Fig. 6o). Together, these findings show that inactivated VEGF/SphK pathway in NP–C PNs causes sphingosine accumulation and this amassment inhibits autophagosome–lysosome fusion by disturbance of calcium homeostasis.

### VEGF rescues autophagic defects in patient-specific cells

To further validate our observation regarding VEGF treatment in NP–C mice, we studied effects of VEGF on SphK activity in human NP–C fibroblasts. Human NP–C fibroblasts cocultured with human BM-MSCs, VEGF^tg^ BM-MSCs or treated with recombinant VEGF showed significantly increased SphK activity, decreased sphingosine and elevated S1P (Supplementary Fig. 7a–c). Increased LC3-II levels and p62 accumulation in NP–C fibroblasts were reduced by VEGF treatment (Supplementary Fig. 7d,e). VEGF-treated NP–C fibroblasts also showed increased calcium release and decreased autophagosome accumulation (as judged by yellow LC3 puncta) compared with non-treated NP–C fibroblasts (Supplementary Fig. 7f,g). Lysosomal exocytosis is necessary to affect clearance of stored intracellular lipids and ameliorates the endolysosomal lipid storage phenotype in NP–C cells^27^. To determine whether VEGF directly induced lysosomal exocytosis, the culture media of normal and NP–C fibroblasts treated with or without VEGF were analysed for the presence of the lysosomal enzyme β-hexosaminidase as a marker for lysosomal content secretion. In all groups, the activity of β-hexosaminidase was not significantly elevated at the indicated times (Supplementary Fig. 7h). The low-level appearance of β-hexosaminidase in the culture media of fibroblasts is not a result of generalized cell lysis, since the levels of lactate dehydrogenase (LDH) in the media remained unchanged in all groups for the duration of the assay (Supplementary Fig. 7h). These results suggested that the ability of VEGF to reduce sphingosine storage in NP–C cells was not due to lysosomal exocytosis.

The recent developments in induced pluripotent stem cells (iPSCs) and iPSC-derived neurons have allowed investigation of pathogenesis of neurological diseases *in vitro*. To explore whether the observed effects of VEGF we describe above were similar in NP–C human neurons, we established human NP–C iPSCs (hNPC-3, 6, 17) by transduction of human NP–C fibroblasts with retroviruses encoding *OCT4*, *SOX2*, *KLF4* and *c-MYC* similar to previous studies^28^,^29^,^30^. Analysis of NP–C iPSCs (hNPC-3) revealed typical characteristics of pluripotent stem cells: similar morphology to embryonic stem cells (ES cells), expression of pluripotent markers including SSEA-4, Tra-1-60 and Tra-1-81, normal chromosomal number and genomic structure, silencing of retroviral transgene and reactivation of genes indicative of pluripotency (Supplementary Fig. 8a–c). The differentiation ability of NP–C iPSCs was also confirmed *in vivo* by teratoma formation (Supplementary Fig. 8d). We analysed SphK activity and sphingolipid levels in the normal iPSC and NP–C iPSC lines. NP–C iPSC lines exhibited decreased SphK activity, increased sphingosine accumulation and decreased S1P levels compared with normal iPSCs (Supplementary Fig. 8e).

**Figure 7:**
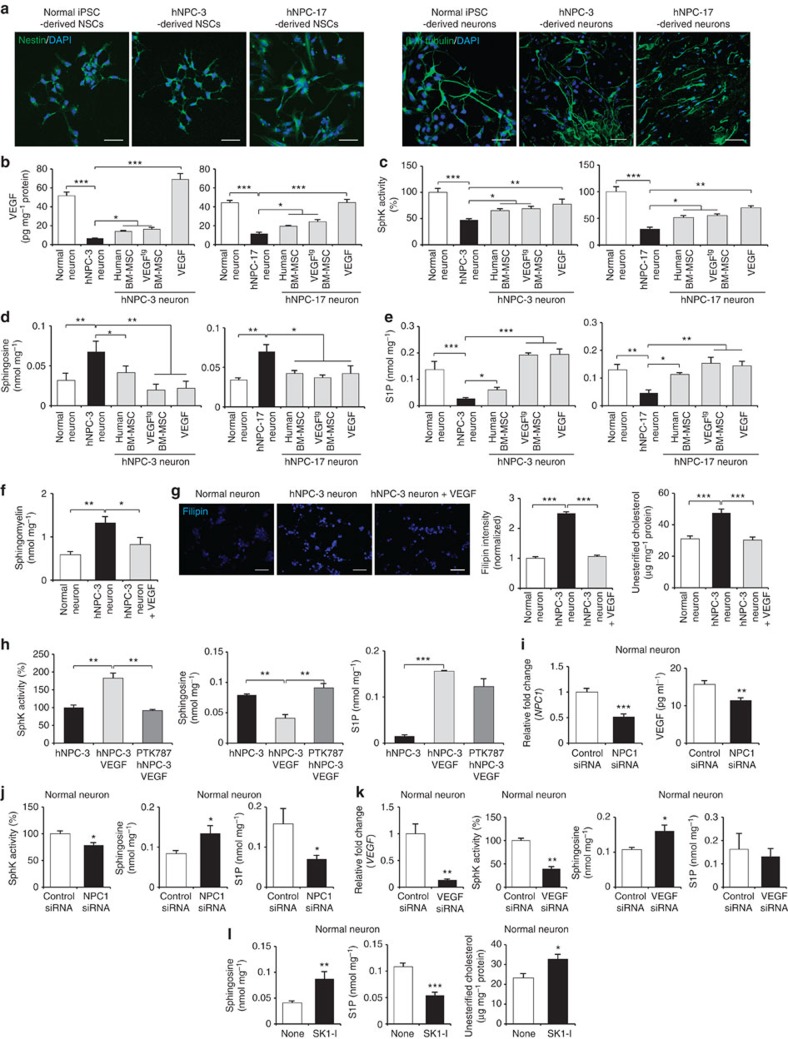
VEGF ameliorates sphingolipid imbalance in NP–C iPSC neurons. (**a**) Left, normal, hNPC-3 and hNPC-17 iPSCs generated nestin-positive neuroprogenitor cells (scale bar, 50 μm). Right, representative images of immunocytochemical staining the β-III tubulin following neural differentiation (scale bar, 50 μm). (**b**) hNPC-3 and hNPC-17 neurons were treated with human BM-MSCs, VEGF^tg^ BM-MSCs or recombinant VEGF (10 ng ml^−1^). Three days after treatment, VEGF levels were measured in cell lysates. (**c**–**f**) SphK activity (**c**), sphingosine (**d**), S1P (**e**) and sphingomyelin (**f**) were measured in normal iPSC neurons and hNPC neurons with or without treatment. (**g**) Filipin staining of unesterified cholesterol in hNPC-3 neurons with or without treatment of recombinant VEGF for 3 days (scale bar, 50 μm). Quantification of filipin fluorescence intensities normalized to normal neurons. Unesterified cholesterol levels in normal iPSC neurons and hNPC neurons with or without treatment were measured (*n*=6 per group). (**h**) Effect of the VEGFR2 inhibitor on VEGF mediated sphingolipid modulation. hNPC-3 neurons were pretreated with PTK787 at 10 μM for 1 day and were treated for 3 days with 10 ng ml^−1^ VEGF and then assayed for SphK activity, sphingosine and S1P (*n*=7 per group). (**i**,**j**) Effect of NPC1 knockdown on sphingolipid factors in normal iPSC neurons. (**i**) Three days after NPC1 siRNA transfection, we measured the levels of *NPC1* mRNA and VEGF expression. (**j**) SphK activity, sphingosine and S1P were measured in normal iPSC neurons treated with control or NPC1 siRNA (control, *n*=7; NPC1 siRNA, *n*=9). (**k**) Effect of VEGF knockdown on sphingolipid factors in normal iPSC neurons. Three days after VEGF siRNA transfection, we measured the levels of *VEGF* mRNA, SphK activity, sphingosine and S1P in normal iPSC neurons (*n*=7 per group). (**l**) Effect of a specific SphK1 inhibitor on sphingolipid factors in normal iPSC neurons. Normal neurons were treated with or without 20 μM SK1-I for 6 h. Lipids were extracted and sphingosine, S1P and unesterified cholesterol levels were determined (*n*=6 per group). **b**–**h**, one-way analysis of variance, Tukey’s *post hoc* test. **i**–**l**, Student’s *t*-test. *P<0.05, **P<0.01, ***P<0.005. All error bars indicate s.e.m.

Next, human neurons were induced from the hNPC-3, hNPC-17 and normal iPSC. Early-differentiating cells expressed nestin and differentiated cells expressed neuron-specific β-III tubulin (Fig. 7a). These NP–C neurons also exhibited phenotypes seen in human NP–C samples, including abnormal VEGF levels and sphingolipid metabolism (Fig. 7b–e). To confirm the effects of VEGF in human NP–C neurons, the hNPC-3- or hNPC-17-derived neurons were cocultured with human or VEGF^tg^ BM-MSCs, or treated with recombinant VEGF. We found that all treated groups exhibited increased VEGF, elevated SphK activity, decreased sphingosine accumulation and increased S1P levels (Fig. 7b–e). Sphingomyelin and unesterified cholesterol levels were also significantly decreased in VEGF-treated NP–C neurons (Fig. 7f,g). We also pretreated NP–C neurons with PTK787 before VEGF treatment. We found that SphK activity and other sphingolipid metabolites in NP–C neurons were mediated by interactions of VEGF and its receptor VEGFR2 in these iPSC-derived NP–C neurons (Fig. 7h).

To reconfirm the *in vitro* mechanism whereby there is a direct relationship between *NPC1*, VEGF and SphK activity in human neurons, we treated normal iPSC neurons with NPC1 and VEGF siRNA (Fig. 7i–k) and determined changes in various sphingolipid factors. NPC1 siRNA decreased VEGF expression and SphK activity (Fig. 7i,j). VEGF siRNA also strongly inactivated SphK levels (Fig. 7k). Both siRNA treatments led to changed levels of sphingosine and S1P, similar to NP–C neurons (Fig. 7j,k). To determine whether reduction in SphK activity affected sphingolipid factors and unesterified cholesterol in iPSC neurons similar to those in classical NP–C cells, we treated the normal iPSC neurons with a specific SphK1 inhibitor, SK1-I. We found that inhibition of SphK activity increased sphingosine and unesterified cholesterol accumulation and decreased cellular S1P (Fig. 7l).

**Figure 8:**
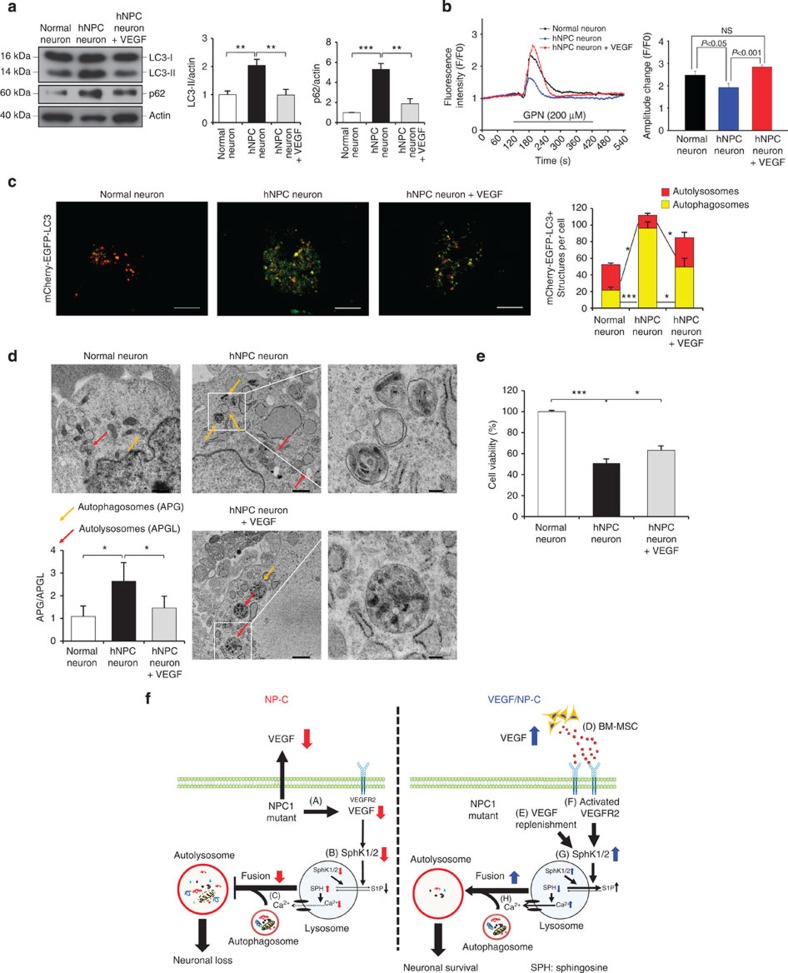
VEGF rescues the autophagic defects in NP–C iPSC neurons. (**a**) Western blot analysis of LC3 and p62 in normal and NP–C iPSC-derived neurons treated with or without 10 ng ml^−1^ recombinant VEGF (normal, *n*=6; hNPC, *n*=7; and VEGF-treated hNPC, *n*=7). (**b**) Left, representative traces showing intracellular [Ca^2+^] changes monitored in single fluo-4-loaded normal and NP–C iPSC neurons treated with or without recombinant VEGF (10 ng ml^−1^). Right, maximal peak fluorescence changes were determined as the differences between basal and the maximum fluorescence, on addition of 200 μM GPN (*n*=10 cells per group). (**c**) Fluorescence staining and quantification of autophagosomes (mCherry^+^-EGFP^+^-LC3) and autolysosomes (mCherry^+^-EGFP^−^-LC3) in normal and NP–C iPSC neurons after recombinant VEGF treatment (normal, *n*=7; hNPC, *n*=8; and VEGF-treated hNPC, *n*=8; scale bar, 10 μm). (**d**) EM images and quantification data of normal and NP–C iPSC-derived neurons after 10 ng ml^−1^ VEGF treatment (*n*=5 per group; low-magnification scale bar, 1 μm; high-magnification scale bar, 200 nm). (**e**) Quantification of cell viability (normal, *n*=5; hNPC, *n*=6; and VEGF-treated hNPC, *n*=6). (**f**) Model of VEGF-mediated SphK activation in NP–C neurons. (A,B) In NP–C cells, sphingosine accumulation is increased due to defective SphK activity together with decreased VEGF caused by mutated NPC1 and defective uptake via VEGFR2. (C) Abnormal sphingosine accumulation decreases calcium release from lysosomes and the reduction in calcium release causes an autophagic defect by inhibiting autophagosome–lysosome fusion. Eventually, these defects cause loss of cerebellar neurons. (D,E) When NP–C neurons are exposed to BM-MSCs or pure VEGF, the cells exhibit elevated intracellular levels of VEGF (F), which induces VEGFR2-mediated activation of SphK in the cytosol and lysosome. (G) This activation leads to decreased sphingosine accumulation and increased S1P levels. (H) Reduced sphingosine accumulation results in improved autophagosome–lysosome fusion by correction of calcium homeostasis. Finally, this restoration prevents neuronal loss in NP–C. **a**–**e**, one-way analysis of variance, Tukey’s *post hoc* test. *P<0.05, **P<0.01, ***P<0.005. All error bars indicate s.e.m.

We also examined whether NP–C neurons exhibited abnormal autophagy. NP–C neurons had significantly higher abnormal autophagic markers than normal neurons (Fig. 8a). VEGF treatment significantly decreased the protein level of abnormal autophagic markers in NP–C neurons (Fig. 8a). Similar to previous results (Supplementary Fig. 7), VEGF-treated NP–C neurons showed increased calcium release and decreased autophagosome accumulation, suggesting that VEGF elevates autophagosome–lysosome fusion (Fig. 8b–d). Consistent with the restored autophagy flux, cell survival was also significantly improved in VEGF-treated NP–C neurons (Fig. 8e). Collectively, these results confirm that defective autophagy by abnormal VEGF/SphK pathway and sphingosine levels in NP–C mice and human fibroblasts also occur in NP–C patient neurons, and replenishment of VEGF is able to ameliorate autophagy defect by correction of sphingolipid imbalance in the NP–C patient cells.

## Discussion

NP–C patients and mice exhibit progressive neuronal loss, mainly of cerebellar PNs, but the mechanism is largely unknown. Recent studies have shown that inactivation of NPC1 caused abnormal autophagy and the defect may contribute to PN loss in NP–C^22^. Loss of NPC1 function leads to trapping of lipids within aberrant membrane compartments, and this may induce a ‘lipid-starvation response’ analogous to the well-characterized autophagic response to amino-acid deprivation^31^. In addition, destructive autophagy in NP–C PNs may also be stimulated hormonally via neurosteroids. Neurosteroids might inhibit autophagy in PNs and when their synthesis is severely decreased, as in NP–C^32^, autophagic cell death might ensue. Similar to previous results^22^,^33^, we found that the impaired autophagic flux in NP–C was associated with decreased autophagosome–lysosome fusion, and that this defect led to PNs loss.

Recent studies have also demonstrated that cholesterol, sphingomyelin and GSL storage are downstream events in NP–C disease pathogenesis caused by sphingosine storage, leading to altered acidic compartment calcium levels^4^. They have determined the chronology of events after inactivation of NPC1. In a drug-induced NP–C cellular model, sphingosine storage in the acidic compartment led to calcium depletion in these organelles, which then resulted in cholesterol, sphingomyelin and GSL storage in these compartments. Therefore, sphingosine storage might be an initiating factor in NPC1 disease pathogenesis that causes altered calcium homeostasis, leading to the secondary storage of sphingolipids and cholesterol, although additional studies are required. Similarly, we found that VEGF-mediated sphingosine modulation also significantly decreased sphingomyelin and unesterified cholesterol levels. Therefore, we suggest that replenishment of VEGF is able to ameliorate accumulation of sphingomyelin and unesterified cholesterol by reducing sphingosine accumulation in the NP–C. Similar to sphingosine accumulation, cholesterol accumulation also induced changes in autophagy–lysosome function in PNs and lead to death of these cells. In NP–C mouse brain, combined LC3 immunofluorescence and filipin staining showed that LC3 accumulated within filipin-labelled cholesterol clusters inside PNs^34^. These results provide strong evidence that cholesterol accumulation-induced changes in autophagy–lysosome function are closely associated with neurodegeneration in NP–C. Therefore, we suggest that reduced PN survival in NP–C may be due to impaired autophagic flux by VEGF/SphK pathway-mediated sphingosine accumulation and secondary storage of cholesterol.

Despite extensive data supporting the modulating effect of VEGF on SphK in NP–C, the clinical effectiveness of VEGF-mediated therapy in the NP–C mouse model was the modest. There are several potential explanations for this finding. First, it must be recognized that in these animal model experiments the primary lesion in the *Npc1* gene and protein remains in the treated mice, and thus correction of their sphingolipid imbalance via VEGF may only slow progression and require additional, combinational therapies to achieve a more complete clinical effect. In addition, VEGF may not be the only factor regulating sphingolipid metabolism in NP–C, as suggested by the fact that the sphingolipid levels were improved, but not normalized, in the treated cells and mice. Finally, the methods used to introduce VEGF into the brain of the NP–C mice may need to be improved, and there are considerable research underway exploring different approaches to introducing proteins such as VEGF into the central nervous system^35^,^36^. Indeed, small molecules may also be developed in the future that modulate VEGFR2 leading to SphK enhancement, or even small molecules that work on SphK directly. Despite the limitations of these animal model studies, however, the findings reported in the manuscript describe a novel pathogenic mechanism in NP–C and reveal a potential approach for the therapy via the VEGF/SphK pathway.

In summary, the data presented here show that VEGF and SphK activities are reduced in both NP–C mouse PNs and patient-specific cells, and that correction of this activity by VEGF (released from BM-MSCs or added directly into the CNS) can reduce NP–C pathological changes via increasing autophagic degradation (Fig. 8f). Thus, VEGF is a therapeutic candidate for NP–C that influences sphingosine storage via SphK modulation, suggesting that enhancing SphK activity is a potential therapeutic intervention for this disorder.

## Methods

### Mice

A colony of Balb/c *Npc1*^nih^ mice has been maintained and the genotype of each mouse was determined by PCR using forward (5′-GGTGCTGGACAGCCAAGTA-3′) and reverse primer (5′-GATGGTCTGTTCTCCCATG-3′)^37^. VEGF-overexpressing transgenic mice^14^ were bred with NP–C mice to generate VEGF/NP–C (*VEGF*^tg^/*Npc1*^−/−^) mice. Four-week-old mice were used for transplantation of BM-MSCs and microspheres. We choose the block randomization method to allocate the animals to experimental groups. For the cerebellar transplantation^7^, the injections were carried out using a glass capillary (1.2 × 0.6 mm). The injection coordinates were 5.52 mm posterior to bregma and injection depth was 2.50 mm. In some experiments, mice were treated with the VEGFR inhibitor PTK787/ZK222584 (PTK787; 100 mg kg^−1^, Selleck Chemicals) or PBS vehicle control by oral gavage once a day for 3 days. PTK787 is a potent and relatively selective inhibitor of all VEGF receptor tyrosine kinases, with greater activity against VEGFR2^17^. Three-week-old WT mice were treated with VEGF shRNA and curcumin (Supplementary Fig. 6a). Eight-week-old male SCID Beige mice (Charles River Laboratories) were used for teratoma formation assay. To eliminate the bias, we were blinded in experimental progress such as data collection and data analysis. Mice were housed at a 12 h day–night cycle with free access to tap water and food pellets. Mouse studies were approved by the Kyungpook National University Institutional Animal Care and Use Committee.

### Cell isolation and culture

Human *NPC1***-**mutant and control fibroblasts (GM03123 and GM05399, respectively) were acquired from the Coriell Institute and were used at passages 10–15. Primary PN cultures were prepared from the cerebellum of individual embryonic day 18 fetuses^7^. The cerebellum was dissociated using the Nerve-Cell Culture System (Sumitomo Bakelite) and plated in the PN culture media^7^. To isolate mouse BM-MSCs^6^, bone marrow was harvested from tibias and femurs of 4- to 6-week-old Balb/c or VEGF-overexpressing transgenic mice, and single-cell suspensions were obtained using a 40-μm cell strainer (BD Biosciences). Cells containing MesenCult MSC Basal Medium plus Supplements (Stemcell Technologies) were plated. Normal iPSC line (HPS0063) was obtained from the RIKEN Bioresource Center^38^. Human BM-MSCs were kindly provided by the Cell Therapy Center of Yonsei University. Informed consent was obtained from all subjects according to the ethics committee guidelines at the Yonsei University Severance Hospital. For some experiments, cells were treated with human recombinant VEGF (R&D Systems), human and mouse SMART pool VEGF siRNA (Dharmacon), human and mouse SMART pool NPC1 siRNA (Dharmacon) or scrambled sequence siRNA control (Dharmacon). NH_4_Cl was used to inhibit autophagic flux. Curcumin (Sigma-Aldrich) was used to increase cytosolic calcium release. For the inhibition of VEGFR2 signal activation *in vitro*, cells were pretreated for 1 day with 10 μM of PTK787. For the inhibition of SphK1 activation *in vitro*, cells were pretreated for 6 h with 20 μM of SK1-I (Enzo Life Sciences).

### Indirect coculture of BM-MSCs

For the indirect coculture experiments, 1.0 μm pore size Millicell Hanging Cell Culture Inserts (Millipore) were placed on top of the previously plated cells. BM-MSCs were seeded onto the insert at a density of 3 × 10^4^ cells per insert. In this system there was no direct contact between cocultured cells and BM-MSCs.

### SphK activity assays

SphK activity was followed as phosphorylation of (7-nitro-2-1,3-benzoxadiazol-4-yl)-d-erythro (NBD)-sphingosine (Avanti Polar Lipids) to NBD-S1P as described previously^39^ with modification using a UPLC (ultra performance liquid chromatography) system (Waters). Quantification was achieved by comparison with NBD-S1P (Avanti Polar Lipids) standards. Cell and tissue lysates were prepared as previously described^7^. Values were expressed as percent of control.

### Lipid extraction and sphingosine/S1P/sphingomyelin quantification

Samples were lysed in homogenization buffer containing 50 mM HEPES (Gibco), 150 mM NaCl (Sigma-Aldrich), 0.2% Igepal (Sigma-Aldrich) and protease inhibitor (Calbiochem)^7^. To quantify the sphingosine, S1P and sphingomyelin levels, the dried lipid extract was resuspended in 0.2% Igepal CA-630. Four microlitres of the lipid extracts was added into 20 μl of NDA derivatization reaction mixture (25 mM borate buffer, pH 9.0, containing 2.5 mM each of NDA and NaCN). The reaction mixture was diluted 1:3 with ethanol, incubated at 50 °C for 10 min and centrifuged (13,000*g* for 5 min). An aliquot (30 μl) of the supernatant was then transferred to a sampling glass vial and 5 μl was applied onto an UPLC system for analysis. The fluorescent sphingosine or S1P derivatives were monitored using a model 474 scanning fluorescence detector (Waters). Quantification of the sphingosine, S1P and sphingomyelin peaks were calculated from sphingosine, S1P and sphingomyelin standard calibration curves using the Waters Millennium software.

### Cytokine antibody array

RayBio Custom Mouse Cytokine Antibody Arrays (RayBiotech) were employed for assay of cell culture supernatants from coculture experiments according to the manufacturer’s instructions.

### ELISA

VEGF protein levels were assayed by using a Mouse and Human VEGF Quantikine kit (R&D Systems) according to manufacturer’s instructions.

### Immunofluorescence staining

For the immunofluorescence staining, cells and brain sections were blocked with PBS containing 5% normal goat serum (Vector Laboratories), 2% BSA (Gibco) and 0.4% Triton X-100 (Sigma-Aldrich). In the same buffer solution, the cells and sections were then incubated for 24 h with primary antibodies. The following antibodies were used: anti-VEGF (rabbit, 1:500, Invitrogen, ab39250), anti-calbindin (rabbit, 1:500, Chemicon, ab82812 and mouse, 1:500, Abcam, ab9481), anti-S1P (mouse, 1:400, Alfresa Pharma, 274594052), anti-LC-3B (rabbit, 1:200, Cell Signaling Technologies, 3868S), anti-active caspase-3 (rabbit, 1:50, Chemicon, AB3623), anti-β-III tubulin (mouse, 1:400, Chemicon, MAB1637), anti-nestin (mouse, 1:400, Chemicon, MAB353), anti-SSEA-4, TRA-1-60 and TRA-1-81 (mouse, 1:100, Chemicon, MAB4304, MAB4360 and MAB4381). The cells and sections were analysed with a laser scanning confocal microscope equipped with Fluoview SV1000 imaging software (Olympus FV1000) or with an Olympus BX51 microscope. Metamorph software (Molecular Devices) was used to calculate the average intensity.

### Filipin staining

Cells and cerebellar sections were fixed with 4% paraformaldehyde for 15 min, washed with PBS and incubated for 30 min with 100 μg ml^−1^ filipin (Polysciences) in PBS. Cells and cerebellar sections were washed twice with PBS for 5 min. The averaged intensities were analysed as described above.

### Amplex red assay

The cells and cerebellar tissues were lysed with lysis buffer (50 mM phosphate buffer, 500 mM NaCl, 25 mM cholic acid and 0.5% Triton X-100). The unesterified cholesterol was determined using the Amplex Red Cholesterol Assay Kit (Molecular Probes) according to the manufacturer’s instructions. After incubation for 30 min at 37 °C, the fluorescence intensities were measured on a microplate reader (Molecular devices) equipped with a filter set for excitation and emission at 560±10 nm and 590±10 nm, respectively. The cholesterol content was calculated with a cholesterol standard curve. Cellular cholesterol content was normalized to protein content.

### GSL analysis

Cerebellum from 6-week-old mice was homogenized with four volumes of ice-cold water in an all-glass Potter-Elvehjem homogenizer; 250 μl of homogenate (50 mg of wet tissue) was extracted by addition of 1.2 ml of methanol and 2 ml of chloroform. After incubation of the samples at 37 °C for 1 h, 1 ml of methanol was added, and the extracts were centrifuged at 2,000 *g* for 10 min. The pellet was re-extracted with 2 ml of chloroform/methanol/water (1/2/0.8, v/v/v) at 37 °C for 2 h. The combined supernatants were concentrated by Speed-Vac, and the dried samples were dissolved in 2 ml of methanol and saponified. After neutralization, samples were diluted with 2 ml of water and desalted using OASIS HLB 1 cc extraction cartridges (Waters). Thin-layer chromatography was performed using HPTLC (Merck) and developed with chloroform/methanol/0.02% CaCl_2_ (5:4:1, v/v). After staining with orcinol–sulfuric acid, GSLs were identified by comparing their *R*_F_ to those of authentic GSL standards.

### Isolation of cytosolic-enriched and lysosome-enriched fractions

Cerebellum from 6-week-old mice was washed twice with cold PBS, and then cytosolic-enriched and lysosome-enriched fractions were extracted. Lysosomes were isolated on sucrose gradient by using a lysosome isolation kit from Sigma-Aldrich. β-*N*-acetyl-glucosaminidase activity quantifications (Sigma-Aldrich), according to the manufacturer instructions, was used to identify lysosomal fractions. Cytosolic-enriched fractions (hydrophilic) were extracted using Mem-PER Membrane Protein Extraction Kit (Pierce) containing protease inhibitor mixture.

### β-Hexosaminidase assays

Human fibroblasts were incubated with recombinant VEGF (10 ng ml^−1^) and 2 mM mannose-6-phosphate at 37 °C. At the indicated time points, an aliquot of media was removed and assayed for β-hexosaminidase activity at 37 °C and pH 4.4 by using the synthetic substrate 4-methylumbelliferyl-*N*-acetyl-glucosaminide (Sigma-Aldrich). After the last time point, cells were lysed and an aliquot of the lysate assayed for β-hexosaminidase activity to determine the total enzyme activity of each sample. Enzyme activities were expressed as a percentage of the total enzyme activity found in the lysate. To confirm cell viability, LDH assays were performed, using an aliquot of culture medium taken at the indicated time points by an LDH assay kit (Sigma-Aldrich).

### Cell viability

Viability of human iPSC-derived neurons was quantified by using WST-1 (Roche). Briefly, human iPSC-derived neurons were seeded on to 24-well plates at a density of 1 × 10^4^ cells per well. Recombinant VEGF (10 ng ml^−1^) was added to culture media, and the cells were incubated for an additional 72 h. WST-1 solution was then added to each well, and the cells were further incubated. After 4 h, the absorbance was measured with a plate reader at 440 nm.

### Laser capture microscopy

LCM was performed by the P.A.L.M. Laser Pressure Catapult system (Zeiss Instruments) using standard procedures. Briefly, cerebella were immediately frozen into a block of tissue freezing medium (Electron Microscopy Sciences). The frozen blocks were cut into 8-μm-thick sections that were then mounted on Arcturus PEN membrane glass slides (Applied Biosystems). The slides were then stained with 0.1% crystal violet (Sigma-Aldrich) and viewed with a Zeiss Observer Z1 inverted light microscope using a × 40 objective. The Zeiss P.A.L.M. device uses a ultraviolet laser beam focused on a selected area of tissue. The collecting cap was placed over the targeted PNs, and by applying a single pulse of laser the targeted cells were catapulted into the collection cap. Total RNA from the isolated cells was extracted with the RNeasy Micro Kit (Qiagen) and then subjected to T7 RNA polymerase-based linear amplification using the Message BOOSTER kit for quantitative PCR (Epicentre).

### Reverse-transcriptase PCR and quantitative real-time PCR

The RNeasy Lipid Tissue Mini kit or RNeasy Plus Mini Kit (Qiagen) was used for extraction of RNA from brain homogenates and cell lysates. Complementary DNA was synthesized from 5 μg of total RNA using the cDNA Synthesis Kit (Clontech) according to the manufacturer’s protocol. Quantitative real-time PCR was performed using a Corbett research RG-6000 real-time PCR instrument. Used primers are described in Supplementary Table 1.

### Behavioural studies

We performed behavioural studies to assess mouse balance and coordination by measuring the amount of time the animal was able to remain on a longitudinally rotating rod. Briefly, the Rota-rod apparatus (Ugo Basile) was set to an initial speed of 4 r.p.m., and the acceleration was increased by 32 r.p.m. every 25–30 s. Scores were registered every 3 days, and three independent tests were performed at each measurement.

### Western blotting

Samples were lysed in RIPA buffer (Cell signaling Technologies), then subjected to SDS–PAGE and transferred to a nitrocellulose membrane. Membranes were blocked with 5% milk, incubated with primary antibody and then incubated with the appropriate horseradish peroxidase-conjugated secondary antibody. Primary antibodies to the following proteins were used: LC3 (rabbit, 1:1,000, 4108S) Beclin-1 (rabbit, 1:1,000, 3738S), p62 (rabbit, 1:1,000, 5114S), rab5 (rabbit, 1:1,000, 3547S), rab7 (rabbit, 1:1,000, 9367S), TFEB (rabbit, 1:1,000, 4240S; all from Cell Signaling Technologies), Lamp1 (rabbit, 1:1,000, Abcam, AB24170), TFEB (rabbit, 1:500, Novus, NBP1-67872), cathepsin D (goat, 1:500, R&D Systems, BAF1029) and β-actin (1:1,000, Santa Cruz, SC-1615). We carried out densitometric quantification using the ImageJ software (US National Institutes of Health). Full scans of western blots are provided in Supplementary Fig. 9.

### Measurement of activity of cathepsin D

Enzyme activity of cathepsin D was determined with cathepsin D activity fluorometric assay kit according to the manufacturer’s protocol (Abcam).

### LysoTracker labelling and quantification

LysoTracker red (Invitrogen) was used at a final concentration of 75 nM. H_2_O_2_- and NH_4_Cl-treated cells were used as positive and negative controls, respectively. The cells were trypsinized, resuspended in PBS and analysed on a FACS Calibur using FACSDiva software (Becton Dickinson).

### Electron microscopy

Brain tissues and cells were fixed in 3% glutaraldehyde/0.1 M phosphate buffer, pH 7.4, and postfixed in 1% osmium tetroxide in Sorensen’s phosphate buffer. After dehydration in ethyl alcohol, the tissues and cells were embedded in Epon (Electron Microscopy Sciences). Samples were cut serially and placed on copper grids and analysed using transmission electron microscope (Tecnai). Images were captured on a digital camera and Xplore3D tomography software.

### Analysis of autophagic flux with mCherry-EGFP-LC3 reporter

mCherry-EGFP-LC3B (plasmid 22418) was acquired from Addgene. Transfection was performed using Lipofectamine2000 (Invitrogen) according to the manufacturer’s protocol. Autophagososome and autolysosome were quantified by image J software.

### Intracellular Ca^2+^ concentration

Changes in [Ca^2+^]_i_ were determined by a confocal laser scanning microscope using a C-apochromat × 40 objective (1.2 numerical aperture). The excitation wavelength for the detection of Ca^2+^ was 488 nm, and the emission wavelength was 516 nm. The fluorescent images were generated at 25 °C and analysed using LSM5 EXCITER software (Carl Zeiss). For the Ca^2+^ measurements, cells were loaded with the Ca^2+^-sensitive dye fluo-4/acetoxymethyl ester (3 μmol l^−1^; Molecular Probes, Eugene, OR, USA) in Krebs-Ringer phosphate-HEPES (KRPH) buffer containing 0.2% BSA (pH 7.4) for 30 min. The cells were incubated for 30 min in a dye-free solution to allow esterase cleavage of the fluo-4/acetoxymethyl ester to liberate fluo-4. After the establishment of a stable baseline [Ca^2+^]_i_ level, the cells were stimulated with 200 μM GPN for 5 min. GPN was applied using a flow system with a flow rate of ~1 ml min^−1^. The images were collected at 5 s intervals, and the results were plotted as the change in fluorescence intensity expressed in arbitrary units. The magnitude was calculated as the change in fluorescence intensity expressed as a percentage of the basal fluorescence intensity (*F*_0_). The area under the curve was calculated using Microcal Origin software version 7.0 (Northampton, MA, USA).

### Lentiviral shRNA-mediated depletion of VEGF and NPC1

We cloned VEGF and NPC1 shRNAs into lentiviral vector plasmid CS-CDF-CG-PRE. The following short hairpin sequences were used: 5′-GATGTGAATGCAGACCAAAGA-3′ (SABiosciences-Qiagen; KM03041N; VEGF-shRNA #4); 5′-AGTTCCAGTACGGCTCCAA-3′ (SABiosciences-Qiagen; KM03041N; NPC1-shRNA #3); and 5′-GGAATCTCATTCGATGCATAC-3′ (SABiosciences-Qiagen; negative control shRNA). The shRNA-expressing lentiviruses were produced by transient transfection of 293T cells^40^. Virus-containing media were collected, filtered and concentrated by ultracentrifugation at 50,000 *g* for 2 h and resuspended in PBS. Viral titres were measured by serial dilution on 293T cells, followed by flow cytometry analysis after 48 h. The titre of the virus used ranged between 2 and 5 × 10^9^ plaque-forming units per ml. Three μl of lentiviruses was administered into the cerebellum of 4-week-old mice by stereotaxic injection 3 days before analysis as previously described^7^.

### Preparation of VEGF-loaded microspheres

VEGF-loaded poly(lactic-co-glycolic acid) (PLGA) microspheres were prepared using the method of water-in-oil-in-water emulsification^41^. Briefly, human recombinant VEGF-A (R&D Systems) in powder form was dispersed in PLGA (50:50 lactic to glycolic acid copolymer ratio with a molecular weight of 40,000–75,000) solution in CH_2_Cl_2_ using a homogenizer. Polyvinylalcohol (PVA) solution (1%) was added to this mixture and homogenized. This emulsion was poured into a 0.1% PVA solution and stirred for 1 h. The hardened microspheres were centrifuged, filtered and washed and subsequently dried for 24 h under vacuum.

### Maintenance and generation of iPSCs

Established iPSC and ES cells were maintained on mitomycin C-treated mouse embryonic fibroblasts (MEFs) in complete ES medium composed of DMEM (Sigma-Aldrich) supplemented with 20% knockout serum replacement, 5 ng ml^−1^ recombinant human basic fibroblast growth factor (FGF) (Peprotech), 20 mM HEPES buffer (pH 7.3), 0.1 mM 2-mercaptoethanol, 0.1 mM non-essential amino acids, 2 mM L-glutamine and 100 U ml^−1^ penicillin/streptomycin (all other materials were from Gibco). NP–C iPSCs were established from NP–C patient skin fibroblasts (GM03123, Coriell Institute)^42^,^43^. In brief, NP–C fibroblasts were seeded at 3 × 10^5^ cells in 60-mm^2^ dish coated with gelatin (Sigma-Aldrich). On day 1, the vesicular stomatitis virus G glycoprotein (VSV-G)-pseudotyped retroviral vector carrying *OCT4*, *SOX2*, *KLF4* and *c-MYC* was added to the fibroblasts. On day 2, cells were subjected to the same transduction procedures and harvested 24 h later. Transduced cells were replated on MEF layers in 100-mm^2^ dish containing the fibroblast medium. On the next day, the medium was changed to complete ES medium with 0.5 mM valproic acid (Sigma-Aldrich), and thereafter changed every other day. After 20 days, ES-like colonies appeared and were picked up to be reseeded on new MEF layers. Cloned ES-like colonies were subjected to further analysis.

### *In vitro* differentiation of human iPSCs

Neural differentiation of iPSCs was performed^44^. Briefly, iPSC colonies were detached from feeder layers and cultured in suspension as embryoid body for about 30 days in bacteriological dishes. EBs were then enzymatically dissociated into single cells and the dissociated cells cultured in suspension in serum-free hormone mix media^44^,^45^ for 10–14 days to allow the formation of neurospheres. Neurospheres were passaged repeatedly by dissociation into single cells, followed by culture in the same manner. Typically, neurospheres between passages 3 and 8 were used for analysis. For terminal differentiation, dissociated neurospheres were allowed to adhere to poly-L-ornithine- and laminin-coated coverslips and cultured for 10 days.

### Alkaline phosphatase staining

Alkaline phosphatase staining was performed using an ES-alkaline phosphatase detection kit (Chemicon) according to manufacturer’s recommendations.

### Teratoma formation and histological analysis

Established iPSCs were prepared at 1 × 10^7^ cells ml^−1^ in PBS. Suspended cells (1–3 × 10^6^) were injected into testes of anaesthetized male SCID Beige mice. Eight weeks after transplantation, mice were sacrificed and tumours were dissected. Tumor samples were fixed in 10% formalin and embedded in paraffin. Sections were stained with hematoxylin and eosin.

### Statistical analysis

Comparisons between two groups were performed with Student’s *t*-test. In cases where more than two groups were compared to each other, a one-way analysis of variance was used, followed by Tukey’s honestly significant difference (HSD) test. Comparisons of overall survival were performed using a log-rank test. All statistical analysis was performed using SPSS statistical software. *P*<0.05 was considered to be significant.

## Author contributions

H.L., J.K.L., M.H.P., Y.R.H., H.K., Y.O., M.O., E.-J.S., J.-H.P., J.-H.B., N.O., X.H. and H.K.J. performed the experiments and analysed the data, J.-S.B. and H.K.J. designed the study and H.L., J.K.L. and J.-S.B. wrote the paper. E.H.S., J.-S.B. and H.K.J. interpreted the data and reviewed the paper. H.H.M. generated and provided VEGF^tg^ mice. All authors discussed results and commented on the manuscript.

## Additional information

**How to cite this article:** Lee, H. *et al.* Pathological roles of the VEGF/SphK pathway in Niemann–Pick type C neurons. *Nat. Commun.* 5:5514 doi: 10.1038/ncomms6514 (2014).

## Supplementary Material

Supplementary Information Supplementary Figures 1-9 and Supplementary Tables 1

## References

[b1] Vanier M. T. & Millat G. Niemann-Pick disease type C. Clin. Genet. 64, 269–281 (2003). 12974729 10.1034/j.1399-0004.2003.00147.x

[b2] Vanier M. T. Lipid changes in Niemann-Pick disease type C brain: personal experience and review of the literature. Neurochem. Res. 24, 481–489 (1999). 10227680 10.1023/a:1022575511354

[b3] Blom T., Li Z., Bittman R., Somerharju P. & Ikonen E. Tracking sphingosine metabolism and transport in sphingolipidoses: NPC1 deficiency as a test case. Traffic 13, 1234–1243 (2012). 22607065 10.1111/j.1600-0854.2012.01379.x

[b4] Lloyd-Evans E. *et al.* Niemann-Pick disease type C1 is a sphingosine storage disease that causes deregulation of lysosomal calcium. Nat. Med. 14, 1247–1255 (2008). 18953351 10.1038/nm.1876

[b5] Bae J. S. *et al.* Bone marrow-derived mesenchymal stem cells promote neuronal networks with functional synaptic transmission after transplantation into mice with neurodegeneration. Stem Cells 25, 1307–1316 (2007). 17470534 10.1634/stemcells.2006-0561

[b6] Bae J. S. *et al.* Neurodegeneration augments the ability of bone marrow-derived mesenchymal stem cells to fuse with Purkinje neurons in Niemann-Pick type C mice. Hum. Gene Ther. 16, 1006–1011 (2005). 16076258 10.1089/hum.2005.16.1006

[b7] Lee H. *et al.* Bone marrow-derived mesenchymal stem cells prevent the loss of Niemann-Pick type C mouse Purkinje neurons by correcting sphingolipid metabolism and increasing sphingosine-1-phosphate. Stem Cells 28, 821–831 (2010). 20201063 10.1002/stem.401

[b8] Le Stunff H., Peterson C., Liu H., Milstien S. & Spiegel S. Sphingosine-1-phosphate and lipid phosphohydrolases. Biochim. Biophys. Acta 1582, 8–17 (2002). 12069805 10.1016/s1388-1981(02)00132-4

[b9] Shu X., Wu W., Mosteller R. D. & Broek D. Sphingosine kinase mediates vascular endothelial growth factor-induced activation of ras and mitogen-activated protein kinases. Mol. Cell. Biol. 22, 7758–7768 (2002). 12391145 10.1128/MCB.22.22.7758-7768.2002 PMC134718

[b10] Xia P. *et al.* Tumor necrosis factor-alpha induces adhesion molecule expression through the sphingosine kinase pathway. Proc. Natl Acad. Sci. USA 95, 14196–14201 (1998). 9826677 10.1073/pnas.95.24.14196 PMC24350

[b11] Olivera A. & Spiegel S. Sphingosine-1-phosphate as second messenger in cell proliferation induced by PDGF and FCS mitogens. Nature 365, 557–560 (1993). 8413613 10.1038/365557a0

[b12] Edsall L. C., Pirianov G. G. & Spiegel S. Involvement of sphingosine 1-phosphate in nerve growth factor-mediated neuronal survival and differentiation. J. Neurosci. 17, 6952–6960 (1997). 9278531 10.1523/JNEUROSCI.17-18-06952.1997 PMC6573266

[b13] Spiegel S. & Milstien S. Sphingosine-1-phosphate: an enigmatic signalling lipid. Nat. Rev. Mol. Cell Biol. 4, 397–407 (2003). 12728273 10.1038/nrm1103

[b14] Wang Y. *et al.* VEGF overexpression induces post-ischaemic neuroprotection, but facilitates haemodynamic steal phenomena. Brain 128, 52–63 (2005). 15509618 10.1093/brain/awh325

[b15] Olsson A. K., Dimberg A., Kreuger J. & Claesson-Welsh L. VEGF receptor signalling - in control of vascular function. Nat. Rev. Mol. Cell Biol. 7, 359–371 (2006). 16633338 10.1038/nrm1911

[b16] Cvetanovic M., Patel J. M., Marti H. H., Kini A. R. & Opal P. Vascular endothelial growth factor ameliorates the ataxic phenotype in a mouse model of spinocerebellar ataxia type 1. Nat. Med. 17, 1445–1447 (2011). 22001907 10.1038/nm.2494 PMC3287040

[b17] Wood J. M. *et al.* PTK787/ZK 222584, a novel and potent inhibitor of vascular endothelial growth factor receptor tyrosine kinases, impairs vascular endothelial growth factor-induced responses and tumor growth after oral administration. Cancer Res. 60, 2178–2189 (2000). 10786682

[b18] Foraker J. E. *et al.* Cross-talk between human mesenchymal stem/progenitor cells (MSCs) and rat hippocampal slices in LPS-stimulated cocultures: the MSCs are activated to secrete prostaglandin E2. J. Neurochem. 119, 1052–1063 (2011). 21954847 10.1111/j.1471-4159.2011.07511.x

[b19] Ruiz de Almodovar C. *et al.* Matrix-binding vascular endothelial growth factor (VEGF) isoforms guide granule cell migration in the cerebellum via VEGF receptor Flk1. J. Neurosci. 30, 15052–15066 (2010). 21068311 10.1523/JNEUROSCI.0477-10.2010 PMC6633861

[b20] Sentilhes L. *et al.* Vascular endothelial growth factor and its high-affinity receptor (VEGFR-2) are highly expressed in the human forebrain and cerebellum during development. J. Neuropathol. Exp. Neurol. 69, 111–128 (2010). 20084021 10.1097/NEN.0b013e3181ccc9a9

[b21] Folkman J. Angiogenesis in cancer, vascular, rheumatoid and other disease. Nat. Med. 1, 27–31 (1995). 7584949 10.1038/nm0195-27

[b22] Ko D. C. *et al.* Cell-autonomous death of cerebellar purkinje neurons with autophagy in Niemann-Pick type C disease. PLoS Genet. 1, 81–95 (2005). 16103921 10.1371/journal.pgen.0010007 PMC1183526

[b23] Rubinsztein D. C. *et al.* In search of an ‘autophagomometer’. Autophagy 5, 585–589 (2009). 19411822 10.4161/auto.5.5.8823

[b24] Settembre C. *et al.* TFEB links autophagy to lysosomal biogenesis. Science 332, 1429–1433 (2011). 21617040 10.1126/science.1204592 PMC3638014

[b25] Pankiv S. *et al.* p62/SQSTM1 binds directly to Atg8/LC3 to facilitate degradation of ubiquitinated protein aggregates by autophagy. J. Biol. Chem. 282, 24131–24145 (2007). 17580304 10.1074/jbc.M702824200

[b26] Bilmen J. G., Khan S. Z., Javed M. H. & Michelangeli F. Inhibition of the SERCA Ca2^+^ pumps by curcumin. Curcumin putatively stabilizes the interaction between the nucleotide-binding and phosphorylation domains in the absence of ATP. Eur. J. Biochem. 268, 6318–6327 (2001). 11733029 10.1046/j.0014-2956.2001.02589.x

[b27] Chen F. W., Li C. & Ioannou Y. A. Cyclodextrin induces calcium-dependent lysosomal exocytosis. PLoS ONE 5, e15054 (2010). 21124786 10.1371/journal.pone.0015054 PMC2993955

[b28] Trilck M. *et al.* Niemann-Pick type C1 patient-specific induced pluripotent stem cells display disease specific hallmarks. Orphanet J. Rare Dis. 8, 144 (2013). 24044630 10.1186/1750-1172-8-144 PMC3848807

[b29] Maetzel D. *et al.* Genetic and chemical correction of cholesterol accumulation and impaired autophagy in hepatic and neural cells derived from Niemann-Pick Type C patient-specific iPS cells. Stem Cell Rep. 2, 866–880 (2014). 10.1016/j.stemcr.2014.03.014 PMC4050353 24936472

[b30] Yu D. *et al.* Niemann-Pick disease type C: induced pluripotent stem cell-derived neuronal cells for modeling neural disease and evaluating drug efficacy. J. Biomol. Screen. 19, 1164–1173 (2014). 24907126 10.1177/1087057114537378 PMC4529815

[b31] Liscum L. & Faust J. R. Low density lipoprotein (LDL)-mediated suppression of cholesterol synthesis and LDL uptake is defective in Niemann-Pick type C fibroblasts. J. Biol. Chem. 262, 17002–17008 (1987). 3680287

[b32] Griffin L. D., Gong W., Verot L. & Mellon S. H. Niemann-Pick type C disease involves disrupted neurosteroidogenesis and responds to allopregnanolone. Nat. Med. 10, 704–711 (2004). 15208706 10.1038/nm1073

[b33] Meske V., Erz J., Priesnitz T. & Ohm T. G. The autophagic defect in Niemann-Pick disease type C neurons differs from somatic cells and reduces neuronal viability. Neurobiol. Dis. 64, 88–97 (2014). 24412309 10.1016/j.nbd.2013.12.018

[b34] Liao G. *et al.* Cholesterol accumulation is associated with lysosomal dysfunction and autophagic stress in Npc1^−/−^ mouse brain. Am. J. Pathol. 171, 962–975 (2007). 17631520 10.2353/ajpath.2007.070052 PMC1959498

[b35] Panyam J. & Labhasetwar V. Biodegradable nanoparticles for drug and gene delivery to cells and tissue. Adv. Drug Deliv. Rev. 55, 329–347 (2003). 12628320 10.1016/s0169-409x(02)00228-4

[b36] Aebischer P. & Ridet J. Recombinant proteins for neurodegenerative diseases: the delivery issue. Trends Neurosci. 24, 533–540 (2001). 11506887 10.1016/s0166-2236(00)01899-3

[b37] Loftus S. K. *et al.* Murine model of Niemann-Pick C disease: mutation in a cholesterol homeostasis gene. Science 277, 232–235 (1997). 9211850 10.1126/science.277.5323.232

[b38] Takahashi K. *et al.* Induction of pluripotent stem cells from adult human fibroblasts by defined factors. Cell 131, 861–872 (2007). 18035408 10.1016/j.cell.2007.11.019

[b39] Billich A. & Ettmayer P. Fluorescence-based assay of sphingosine kinases. Anal. Biochem. 324, 114–119 (2004). 14769343 10.1016/j.ab.2003.11.018

[b40] Miyoshi H., Blomer U., Takahashi M., Gage F. H. & Verma I. M. Development of a self-inactivating lentivirus vector. J. Virol. 72, 8150–8157 (1998). 9733856 10.1128/jvi.72.10.8150-8157.1998 PMC110156

[b41] Kim T. K. & Burgess D. J. Pharmacokinetic characterization of 14C-vascular endothelial growth factor controlled release microspheres using a rat model. J. Pharm. Pharmacol. 54, 897–905 (2002). 12162707 10.1211/002235702760089009

[b42] Okita K., Ichisaka T. & Yamanaka S. Generation of germline-competent induced pluripotent stem cells. Nature 448, 313–317 (2007). 17554338 10.1038/nature05934

[b43] Okabe M. *et al.* Definitive proof for direct reprogramming of hematopoietic cells to pluripotency. Blood 114, 1764–1767 (2009). 19564635 10.1182/blood-2009-02-203695

[b44] Okada Y. *et al.* Spatiotemporal recapitulation of central nervous system development by murine embryonic stem cell-derived neural stem/progenitor cells. Stem Cells 26, 3086–3098 (2008). 18757299 10.1634/stemcells.2008-0293

[b45] Imaizumi Y. *et al.* Mitochondrial dysfunction associated with increased oxidative stress and alpha-synuclein accumulation in PARK2 iPSC-derived neurons and postmortem brain tissue. Mol. Brain 5, 35 (2012). 23039195 10.1186/1756-6606-5-35 PMC3546866

