# Supplementary material for: Pathological roles of the VEGF/SphK pathway in Niemann–Pick type C neurons
Source: Nat Commun. 2014 Nov 24;5:5514. doi: 10.1038/ncomms6514 (PMC4263144; doi:10.1038/ncomms6514)
Supplement: Supplementary Information — Supplementary Figures 1-9 and Supplementary Tables 1 [file ncomms6514-s1.pdf]

Supplementary Information for

Pathological roles of the VEGF/SphK pathway in Niemann-Pick Type C neurons

Hyun Lee, Jong Kil Lee, Min Hee Park, Yu Ri Hong, Hugo H. Marti, Hyongbum Kim, Yohei Okada, Makoto Otsu, Eul-Ju Seo, Jae-Hyung Park, Jae-Hoon Bae, Nozomu Okino, Xingxuan He, Edward H Schuchman, Jae-sung Bae and Hee Kyung Jin

The PDF file includes:

Supplementary Table 1 Sequences of primer pairs
Supplementary Figure 1: Soluble VEGF derived from BM-MSCs cocultured with NP-C PNs.
Supplementary Figure 2: VEGF derived from BM-MSCs modulates abnormal sphingolipids in NP-C PNs.
Supplementary Figure 3: VEGF derived from BM-MSCs restores sphingolipid imbalance in NP-C mice.
Supplementary Figure 4: Genetic VEGF overexpression decreases sphingosine and increases S1P levels in NP-C mice.
Supplementary Figure 5: Intracerebellar delivery of recombinant VEGF using microsphere restores sphingolipid imbalance in NP-C mice.
Supplementary Figure 6: Curcumin reduces the autophagic defects caused by VEGF-mediated sphingosine accumulation.
Supplementary Figure 7: VEGF-mediated restoration of sphingolipid imbalance and autophagy defect in NP-C fibroblast.
Supplementary Figure 8: Generation of NP-C iPSCs from patient fibroblasts.
Supplementary Figure 9: Uncropped images of western blots included in figures.

Supplementary Table 1 Sequences of primer pairs

Gene	Forward	Reverse
Vegf	5'-CTGTGCAGGCTGCTGTAACG-3'	5'-GTTCCCGAAACCCTGAGGAG-3'
VEGFR2	5'-GCCCTGCTGTGGTCTTAC-3'	5'-CAAAGCATTGCCCATTCGAT-3'
Sphk1	5'-GGCTCTGCAGCTCTTCCAGAG-3'	5'-CTCCTCTGCACACACCAGCTC-3'
Gabra6	5'-TGACTTGGACTGATGAGAGAC-3'	5'-GGATAAGCATAGCTCCCAAAC-3'
Npc1	5'-GTCTTACTCGGAGCCACTCA-3'	5'-AAATTGAGGAGCCGTTCTCT-3'
Gapdh	5'-ACAACTTTGGCATTGTGGAA-3'	5'-GATGCAGGGATGATGTTCTG-3'
NPC1	5'-CAGGCCTACCAGAGAGATGA-3'	5'-CGGACAATGCTCCTAAGAAA-3'
VEGF	5'-TTTTGGAAACCAGCAGAAAG-3'	5'-CCAAAAGCAGGTCACTCACT-3'
NODAL	5'-GGGCAAGAGGCACCGTCGACATCA-3'	5'-GGGACTCGGTGGGGCTGGTAACGTTTC-3'
GDF3	5'-CTTATGCTACGTAAAGGAGCTGGG-3'	5'-GTGCCAACCCAGGTCCCGGAAGTT-3'
LEFTY1	5'-CTTGGGGACTATGGAGCTCAGGGCGAC-3'	5'-CATGGGCAGCGAGTCAGTCTCCGAGG-3'
LEFTY2	5'-GCTGGAGCTGCACACCCTGGACCTCAG-3'	5'-GGGCAGCGAGGCAGTCTCCGAGGC-3'
DPPA2	5'-CCGTCCCCGCAATCTCCTTCCATC-3'	5'-ATGATGCCAACATGGCTCCCGGTG-3'
ESG1	5'-ATATCCCGCCGTGGGTGAAAGTTC-3'	5'-ACTCAGCCATGGACTGGAGCATCC-3'
TDGF1	5'-CTGCTGCCTGAATGGGGGAACCTGC-3'	5'-GCCACGAGGTGCTCATCCATCACAAGG-3'
SOX2	5'-GGGAAATGGGAGGGGTGCAAAAGAGG-3'	5'-TTGCGTGAGTGTGGATGGGATTGGTG-3'
NANOG	5'-CAGCCC**C**GATTCTTCCACCAGTCCC -3'	5'-CGGAAGATTCCCAGTCGGGTTCACC-3'
ZFP42	5'-CAGATCCTAAACAGCTCGCAGAAT-3'	5'-GCGTACGCAAATTAAAGTCCAGA-3'
UTF1	5'-CCGTCGCTGAACACCGCCCTGCTG-3'	5'-CGCGCTGCCCAGAATGAAGCCCAC-3'
DNMT3B	5'-TGCTGCTCACAGGGCCCGATACTTC-3'	5'-TCCTTTCGAGCTCAGTGCACCACAAAAC-3'
OCT3/4 tg	5'-CAACGAGAGGATTTTGAGGCT-3'	5'-TACAGGTGGGGTCTTTACTTC-3'
SOX2 tg	5'-TGCAGTACAACTCCATGACCA-3'	5'-TACAGGTGGGGTCTTTACTTC-3'
KLF4 tg	5'-TGCGGCAAAACCTACACAAAG-3'	5'-TACAGGTGGGGTCTTTCATTC-3'
c-MYC tg	5'-CAACAACCGAAAATGCACCAGCCCCAG-3'	5'-TACAGGTGGGGTCTTTCATTC-3'
DPPA4	5'-GGAGCCGCCTGCCCTGGAAAATTC-3'	5'-TTTTTCCTGATATTCTATTCCCAT-3'
GAPDH	5'-AACAGCCTCAAGATCATCAGC-3'	5'-TTGGCAGGTTTTTCTAGACGG-3'

a

Mouse cytokine antibody array panel

	A	B	C	D	E	F	G	H
1	Positive	Positive	Negative		EGF	IGFBP-3	MIP-1α	IL-4
2	MIP-2	bFGF	IGF-1	IL-6	IL-10	TNF-α	IFN-γ	Fractalkine
3	IL-17	VEGF	RANTES	IL-1β	MCP-1	IL-2	SDF-1α	Shh-N

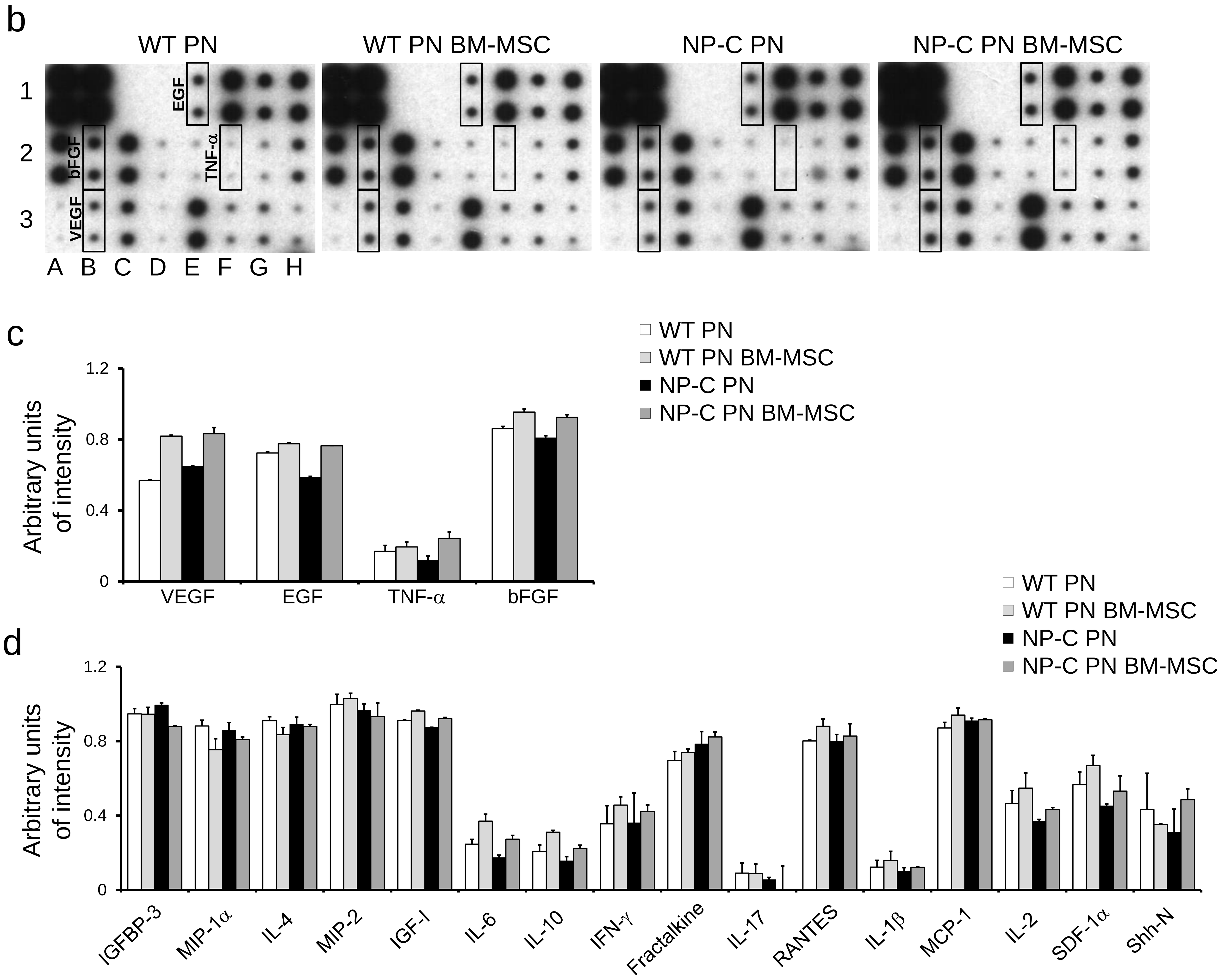

Supplementary Figure 1: Soluble VEGF derived from BM-MSCs cocultured with NP-C PNs. The cytokine antibody array was performed using CM derived from PNs with or without BM-MSCs. (**a**) The names and locations of each cytokine/chemokine custom spot for this set of experiments are listed. (**b**) The boxed areas indicate up-regulated proteins in the PNs CM after BM-MSCs coculture. (**c**) Films were scanned and analyzed using the Bio-Rad analysis software. The optical intensity of the cytokine spots of interest (boxed) was quantified. (**d**) The average optical intensity for each pair of listed cytokine spots is shown. Error bars indicate s.e.m.

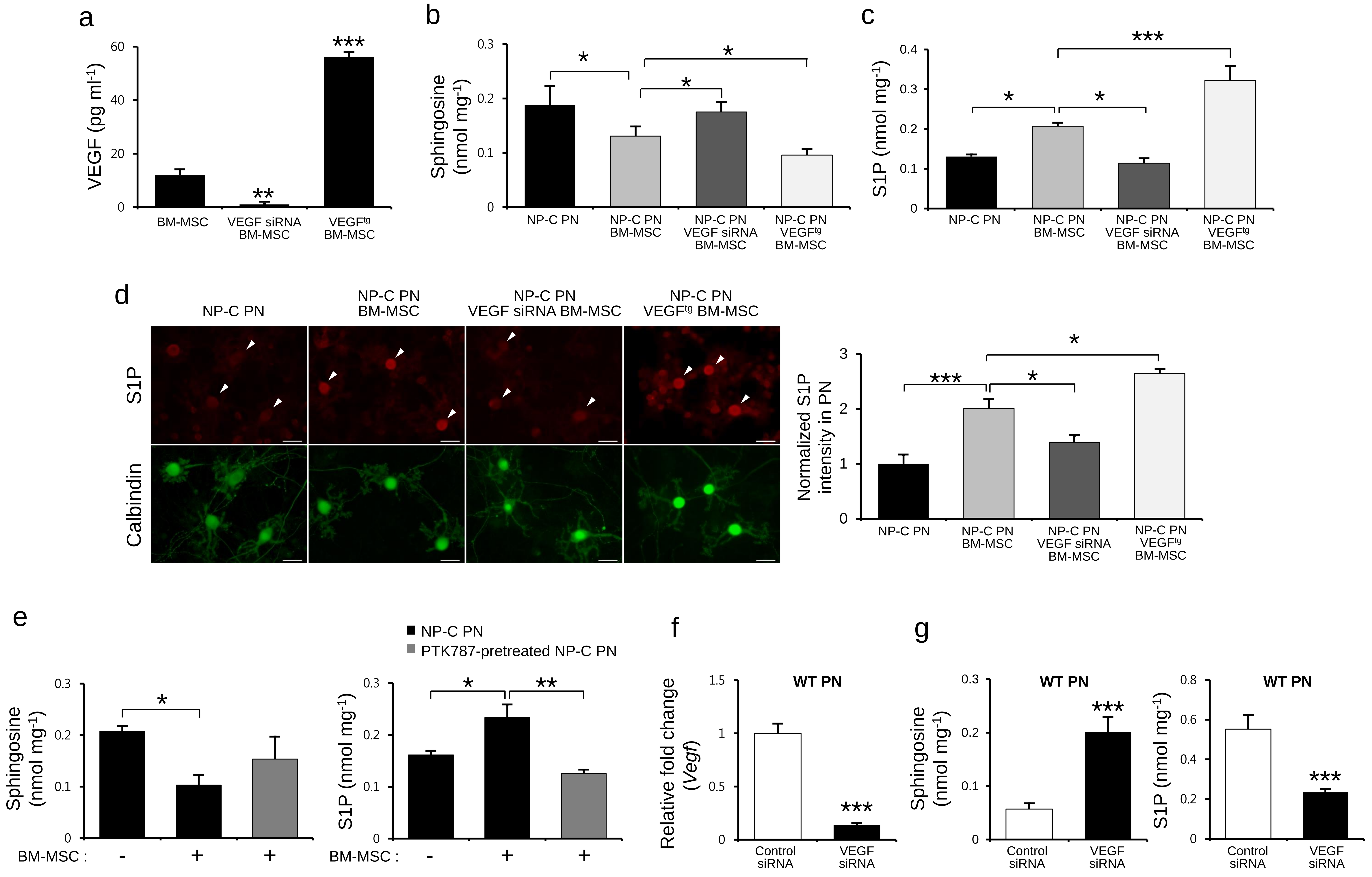

Supplementary Figure 2: VEGF derived from BM-MSCs modulates abnormal sphingolipids in NP-C PNs. (**a**) The levels of VEGF were assayed in the CM of normal BM-MSCs, VEGF siRNA BM-MSCs and VEGF^{tg} BM-MSCs (*n* = 7 per group). (**b,c**) Sphingosine (**b**) and S1P (**c**) were measured in NP-C PNs alone and NP-C PNs cocultured with normal BM-MSCs, VEGF siRNA BM-MSCs and VEGF^{tg} BM-MSCs (*n* = 8 per group). (**d**) PNs were costained with anti-calbindin and anti-S1P (scale bar, 50 μm). Arrowheads indicate S1P expression by PNs. Values represent normalized fluorescence intensities of S1P in PNs (*n* = 8 per group). (**e**) Effect of the VEGFR2 inhibition on BM-MSCs mediated sphingolipid modulation. NP-C PNs were pretreated with PTK787 at 10 μM for 1 day and were cocultured for 3 days with BM-MSCs and then measured for sphingosine and S1P (NP-C PNs, *n* = 7; NP-C PNs cocultured with BM-MSCs, *n* = 8; and PTK787 pretreated NP-C PNs cocultured with BM-MSCs, *n* = 8). (**f,g**) Effect of VEGF knockdown on sphingolipid factors in PNs. Three days after VEGF siRNA transfection, we measured the levels of *Vegf* mRNA (**f**), sphingosine and S1P (**g**) in PNs (control, *n* = 6; and VEGF siRNA, *n* =8). a-e, one-way ANOVA, Tukey's post hoc test. f and g, Student's *t* test. *$P < 0.05$, **$P < 0.01$, ***$P < 0.005$. All error bars indicate s.e.m.

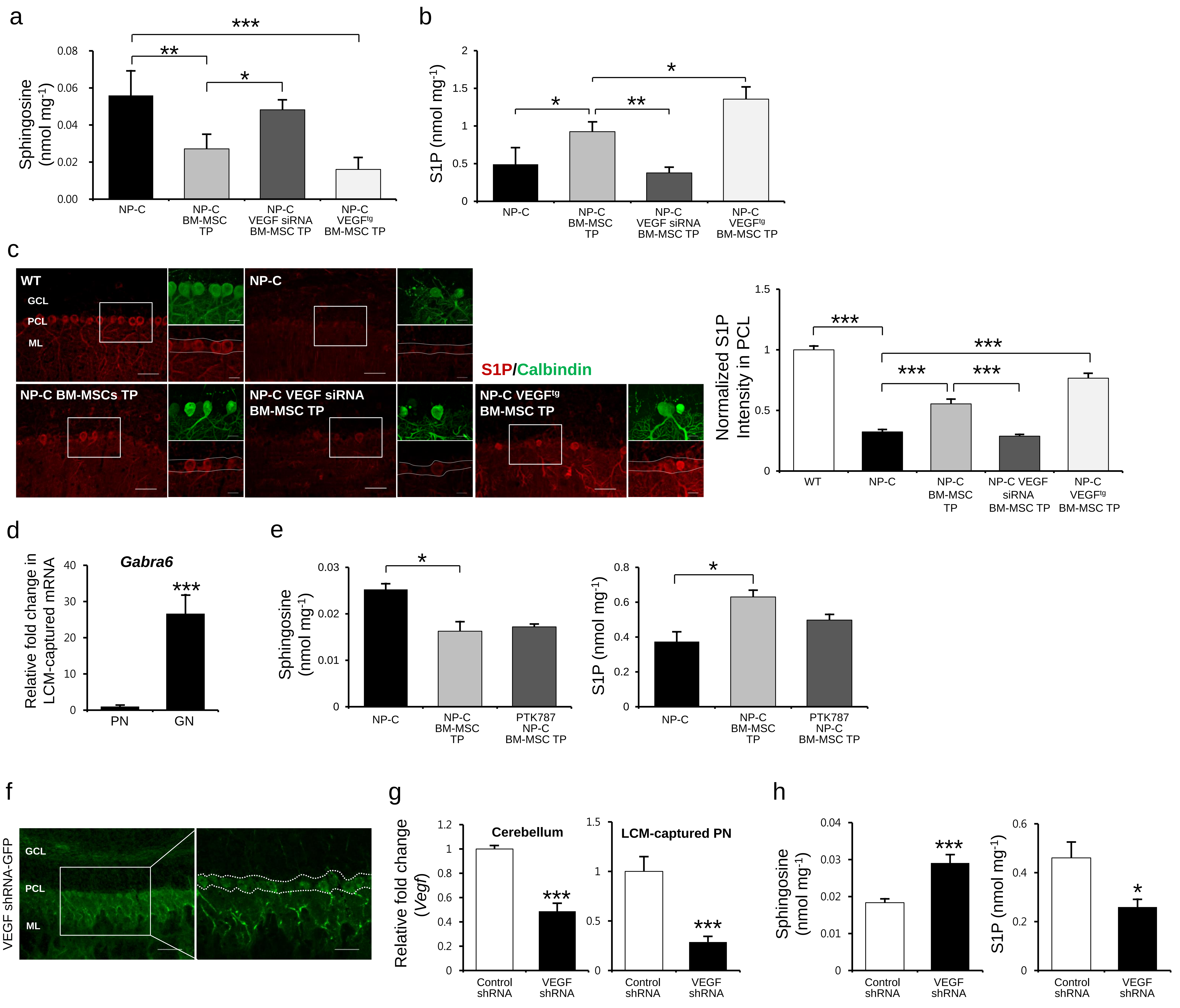

Supplementary Figure 3: VEGF derived from BM-MSCs restores sphingolipid imbalance in NP-C mice. (**a**,**b**) Sphingosine (**a**) and S1P (**b**) were measured in the cerebellums of NP-C mice treated with PBS, BM-MSCs, VEGF siRNA BM-MSCs and VEGF^{tg} BM-MSCs (*n* = 8 for each group). (**c**) Two weeks after transplantation, cerebellar sections were stained with anti-calbindin and anti-S1P (Low magnification scale bar, 50 μm; high magnification scale bar, 20 μm). Values represent normalized S1P fluorescence intensities in PCL (*n* = 8 brains and *n* = 15 sections in each group). (**d**) Cell purity after LCM. LCM samples of PNs were analyzed by quantitative real-time PCR for GN contamination by testing for the GN-specific marker *Gabra6*, in comparison to LCM samples of GN. (**e**) NP-C mice were treated daily with the PTK787 at 100 mg/kg or PBS vehicle control, starting 2 days prior to the BM-MSCs transplantation. One day after BM-MSCs treatment, sphingosine and S1P were measured (NP-C, *n* = 7; BM-MSCs transplanted NP-C, *n* = 8; and BM-MSCs transplanted NP-C with PTK787 pretreatment, *n* = 8). (**f**) Images of GFP in mice cerebellum show the efficiency of VEGF shRNA-GFP gene transfer (scale bar, 50 μm). (**g**,**h**) Effect of VEGF knockdown on sphingolipid factors in cerebellums. After intracerebellar injection of mice with control or VEGF shRNA, relative levels of *Vegf* mRNA (**g**), sphingosine and S1P (**h**) were measured (control, *n* = 7; and VEGF shRNA, *n* =8). a-c and e, one-way ANOVA, Tukey's post hoc test. d, g and h, Student's *t* test. $*P < 0.05$, $**P < 0.01$, $***P < 0.005$. All error bars indicate s.e.m.

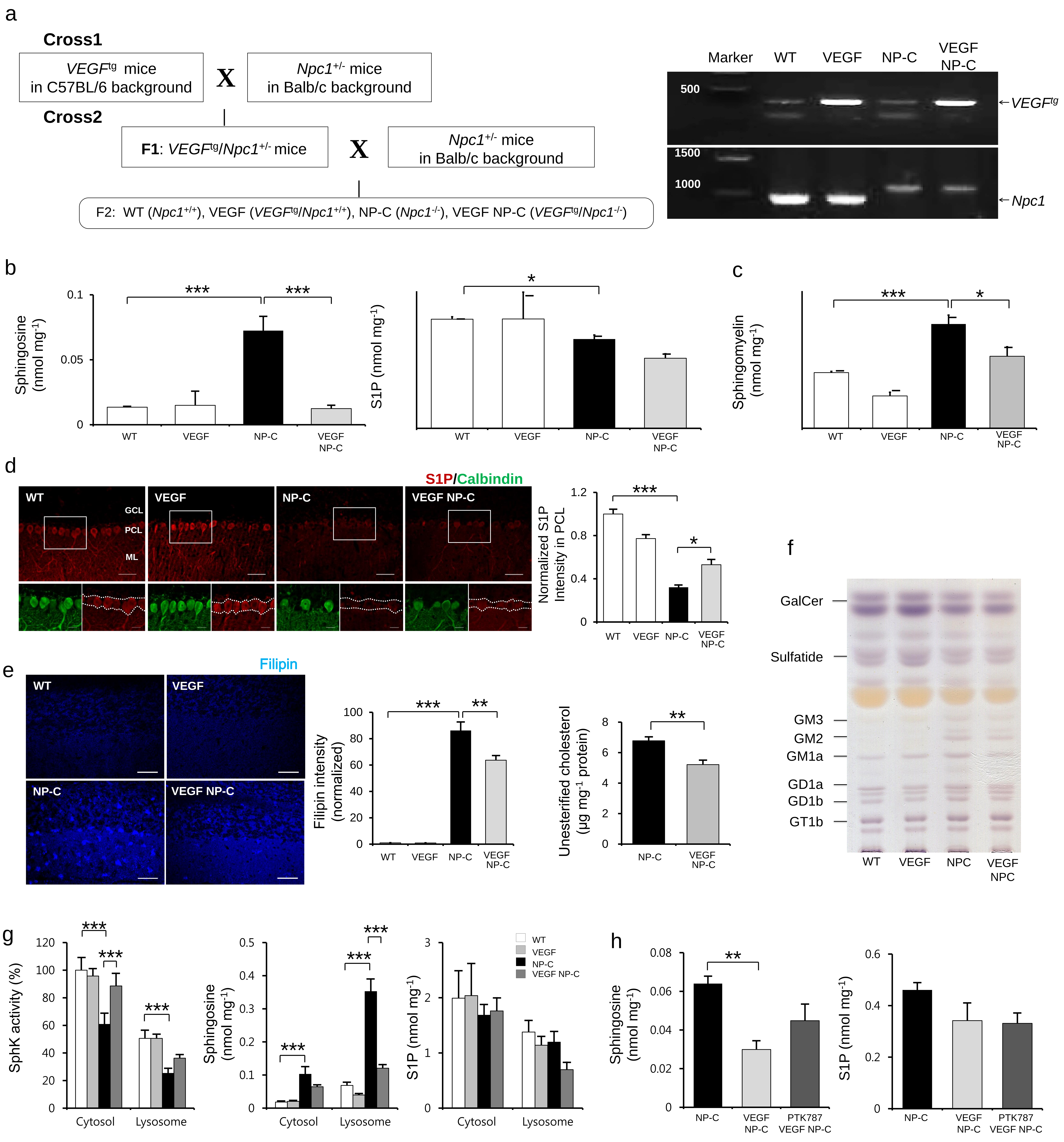

Supplementary Figure 4: Genetic VEGF overexpression decreases sphingosine and increases S1P levels in NP-C mice. (**a**) Crossing scheme to generate WT, VEGF, NP-C and VEGF/NP-C mice. PCR-based detection of genotypes in mice. (**b**) Sphingosine and S1P were measured in cerebellums from 6-week-old WT, VEGF, NP-C and VEGF/NP-C mice (*n* = 8 per group). (**c**) Sphingomyelin was measured in cerebellums from 6-week-old WT, VEGF, NP-C and VEGF/NP-C mice (*n* = 8 per group). (**d**) Cerebellar sections were stained with anti-calbindin and anti-S1P (Low magnification scale bar, 50 µm; high magnification scale bar, 20 µm). Values represent normalized S1P fluorescence intensities in PCL (*n* = 7 brains and *n* = 15 sections in each group). (**e**) Cerebellar sections were stained with filipin (scale bar, 50 µm). Quantification of filipin fluorescence intensities normalized to WT mice (*n* = 5 brains and *n* = 5 sections in each group). Unesterified cholesterol levels were measured in cerebellums from 6-week-old NP-C and VEGF/NP-C mice (*n* = 7 per group). (**f**) GSL patterns in cerebellum of 6-week-old WT, VEGF, NP-C and VEGF/NP-C mice (*n* = 3 per group). Abnormally high levels of GM2 and GM3 were observed in brains of NP-C mice, compared to brains of WT mice. Although the ratios differed slightly, the GSL pattern of VEGF/NP-C mice resembled that of NP-C mice. (**g**) The cytosolic-enriched and lysosome-enriched fractions were isolated from cerebellum. The levels of SphK activity, sphingosine and S1P in these isolated fractions were determined (*n* = 8 per group). (**h**) VEGF/NP-C mice were treated daily with the PTK787 at 100 mg/kg or PBS vehicle control for 3 days before sacrifice (6-week-old), and sphingosine and S1P were measured (*n* = 8 per group). b-d, e (left), g and h, one-way ANOVA, Tukey's post hoc test. e (right), Student's *t* test. *$P < 0.05$, **$P < 0.01$, ***$P < 0.005$. All error bars indicate s.e.m.

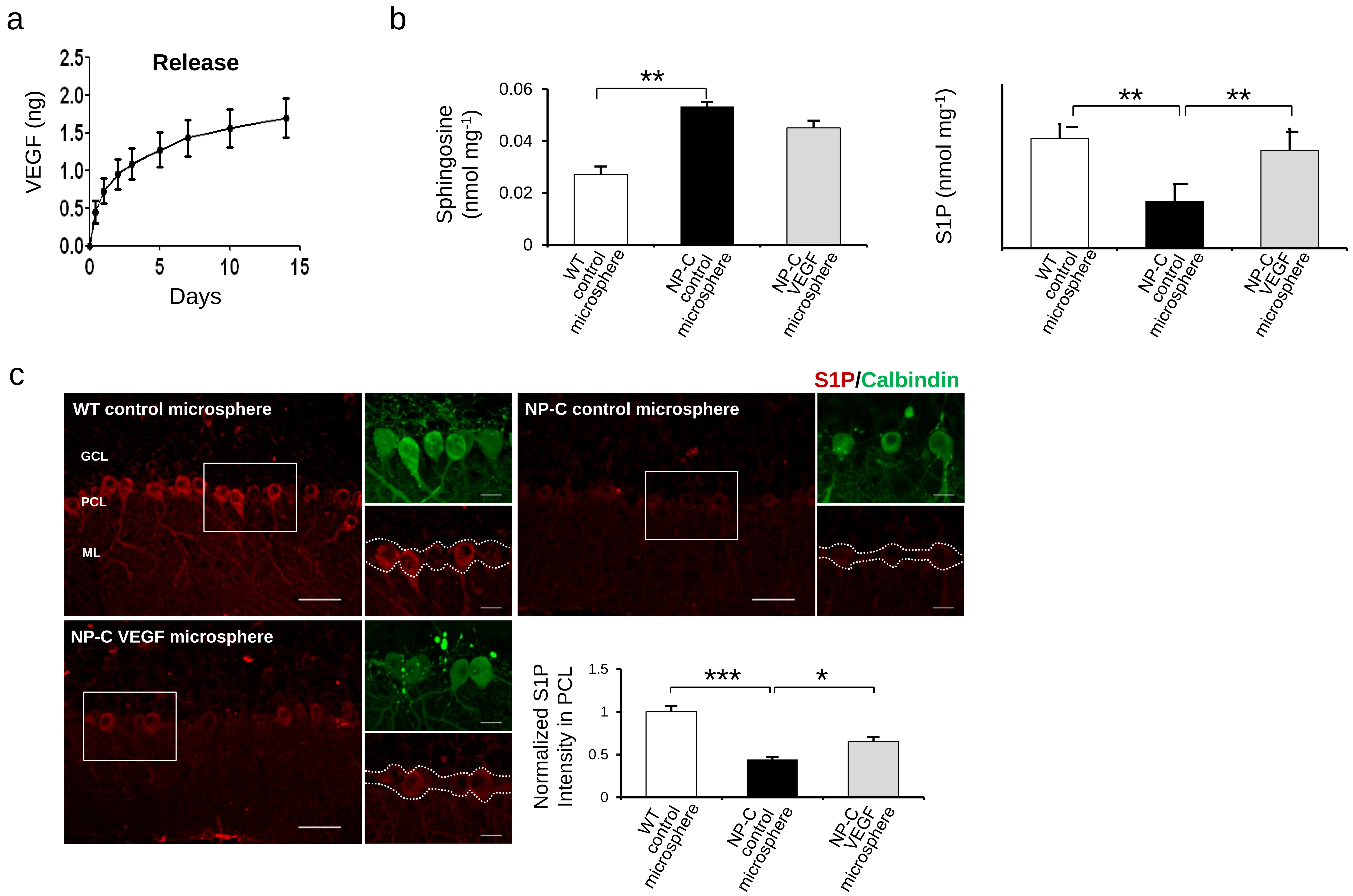

Supplementary Figure 5: Intracerebellar delivery of recombinant VEGF using microsphere restores sphingolipid imbalance in NP-C mice. (**a**) Cumulative VEGF release over 2 weeks from 1 mg of VEGF-loaded microspheres was determined by ELISA (n = 7 per group). (**b**) Sphingosine and S1P were estimated in the cerebellums of WT and NP-C mice at one day after control or VEGF-loaded microsphere treatment (n = 7 per group). (**c**) Cerebellar sections were prepared at 2 weeks after transplantation and immunostained with anti-calbindin and anti-S1P (Low magnification scale bar, 50 μm; high magnification scale bar, 20 μm). Values represent normalized S1P fluorescence intensities in PCL (n = 7 brains and n = 15 sections in each group). b and c one-way ANOVA, Tukey's post hoc test. *$P < 0.05$, **$P < 0.01$, ***$P < 0.005$. All error bars indicate s.e.m.

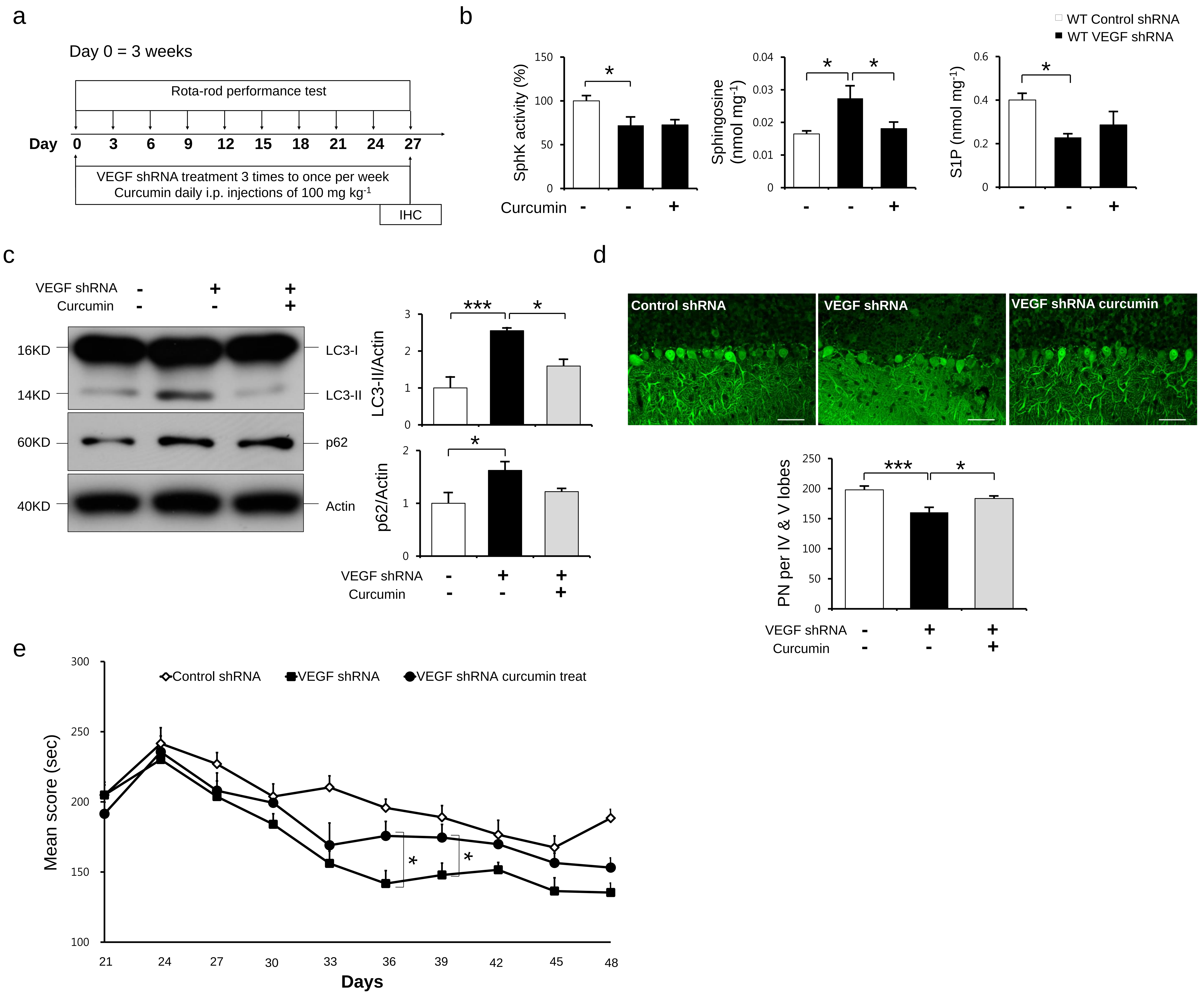

Supplementary Figure 6: Curcumin reduces the autophagic defects caused by VEGF-mediated sphingosine accumulation. (**a**) Protocol of VEGF knockdown and curcumin treatment. (**b**) Sphk activity, sphingosine and S1P levels were measured in control and VEGF shRNA transfected mice cerebellums treated with or without curcumin (control, n = 6; VEGF shRNA, n = 7; and VEGF shRNA/curcumin, n = 7). (**c**) Western blot analysis of LC3 and p62 in control and VEGF shRNA transfected mice cerebellums treated with or without curcumin (control, n = 6; VEGF shRNA, n = 8; and VEGF shRNA/curcumin, n = 8). (**d**) Cerebellar sections were stained with anti-calbindin (scale bar, 50 μm), and the number of calbindin-positive PNs were quantified (control, n = 6; VEGF shRNA, n = 7; and VEGF shRNA/curcumin, n = 7). (**e**) Rota-Rod scores of mice were averaged and plotted (n = 8 per group). b-e, one-way ANOVA, Tukey's post hoc test. *$P < 0.05$, ***$P < 0.005$. All error bars indicate s.e.m.

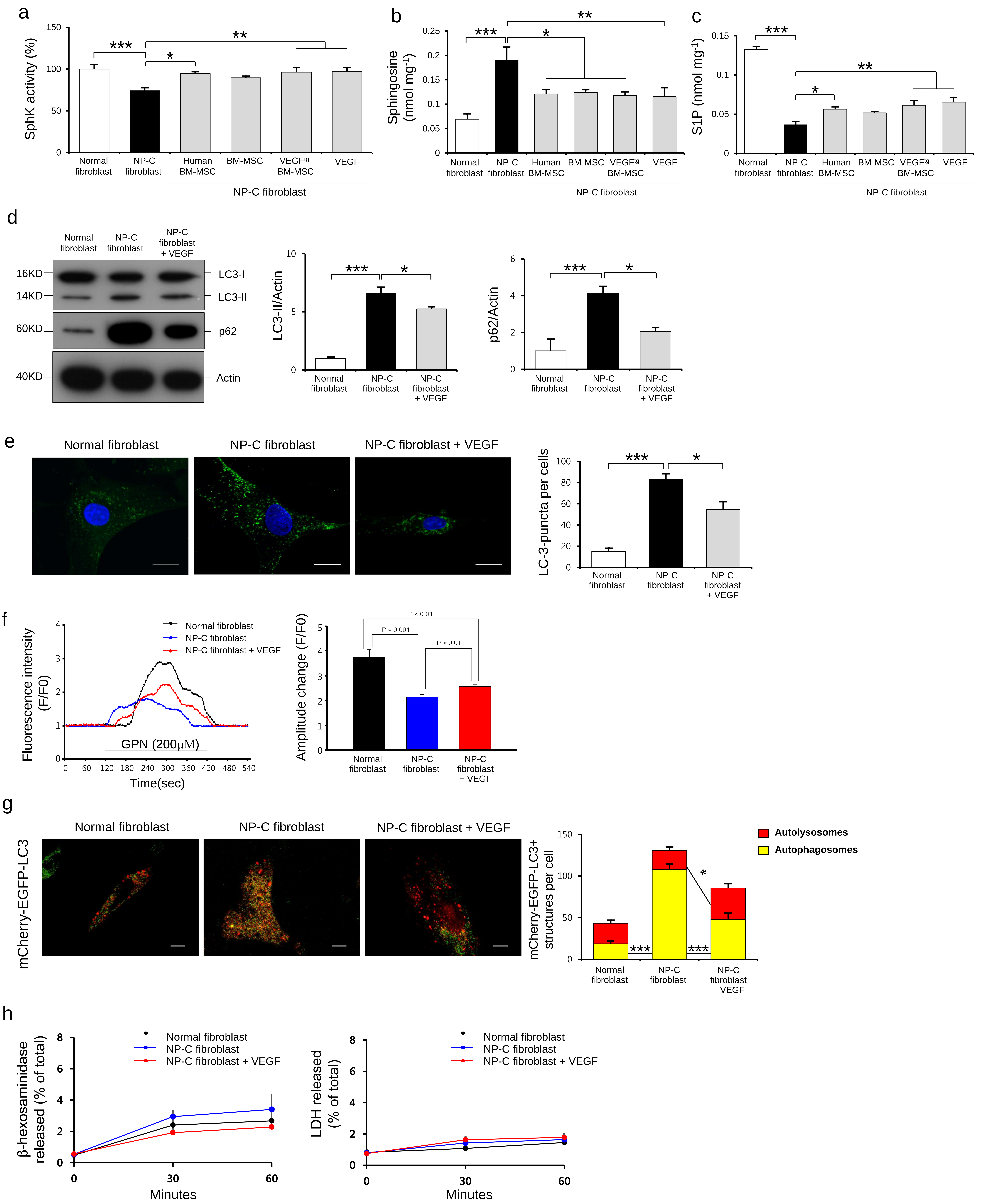

a
SphK activity (%)
Normal fibroblast
NP-C fibroblast
Human BM-MSC
BM-MSC
VEGF^tg BM-MSC
VEGF
NP-C fibroblast
b
Sphingosine (nmol mg-1)
c
S1P (nmol mg-1)
d
Normal fibroblast
NP-C fibroblast
NP-C fibroblast + VEGF
16KD
14KD
60KD
40KD
LC3-I
LC3-II
p62
Actin
LC3-II/Actin
p62/Actin
e
LC-3-puncta per cells
f
Fluorescence intensity (F/F0)
GPN (200μM)
Time(sec)
Amplitude change (F/F0)
P < 0.01
P < 0.001
g
mCherry-EGFP-LC3
mCherry-EGFP-LC3+ structures per cell
Autolysosomes
Autophagosomes
h
β-hexosaminidase released (% of total)
LDH released (% of total)
Minutes

Supplementary Figure 7: VEGF-mediated restoration of sphingolipid imbalance and autophagy defect in NP-C fibroblast. (**a**) SphK activity was measured in normal fibroblast (*n* = 7) and NP-C patient fibroblasts treated with human BM-MSCs, BM-MSCs, VEGF^{tg} BM-MSCs and 10 ng/ml recombinant VEGF (*n* = 8 per group). (**b,c**) Sphingosine (**b**) and S1P (**c**) levels were measured using UPLC system in normal fibroblast (*n* = 7) and NP-C patient fibroblasts treated with human BM-MSCs, BM-MSCs, VEGF^{tg} BM-MSCs and 10 ng/ml recombinant VEGF (*n* = 8 per group). (**d**) Western blot analysis of LC3 and p62 in normal fibroblast (*n* = 6) and NP-C patient fibroblasts treated with or without 10 ng/ml recombinant VEGF (*n* = 7 per group). (**e**) Immunocytochemistry of LC3 in normal fibroblast and NP-C patient fibroblasts treated with or without recombinant VEGF (scale bar, 10 μm, *n* = 6 per group). (**f**) Left, representative traces showing intracellular [Ca^{2+}] changes monitored in single fluo-4-loaded human fibroblasts. The data are presented as the normalized changes in the fluorescence from the basal values (F/F_0; fluorescence/fluorescence at time 0). Lysosomal calcium content was assessed upon addition of 200 μM GPN. Right, maximal peak fluorescence changes were determined as the differences between basal and the maximum fluorescence, upon addition of 200 μM GPN (*n* = 10 cells per group). **(g)** Fluorescence staining and quantification of autophagosomes (mCherry^{+}-EGFP^{+}-LC3) and autolysosomes (mCherry^{+}-EGFP^{-}-LC3) in normal fibroblast (*n* = 6) and NP-C patient fibroblasts treated with or without 10 ng/ml recombinant VEGF (*n* = 8 per group, scale bar, 10 μm). **(h)** Normal fibroblast (*n* = 6) and NP-C fibroblasts (*n* = 8 per group) incubated with or without 10 ng/ml recombinant VEGF. At the indicated times, an aliquot of media was assayed for β-hexosaminidase and LDH activity. All enzyme activities are expressed as the percentage of the total enzyme activity found in cells. a-h one-way ANOVA, Tukey's post hoc test. *$P < 0.05$, **$P < 0.01$, ***$P < 0.005$. All error bars indicate s.e.m.

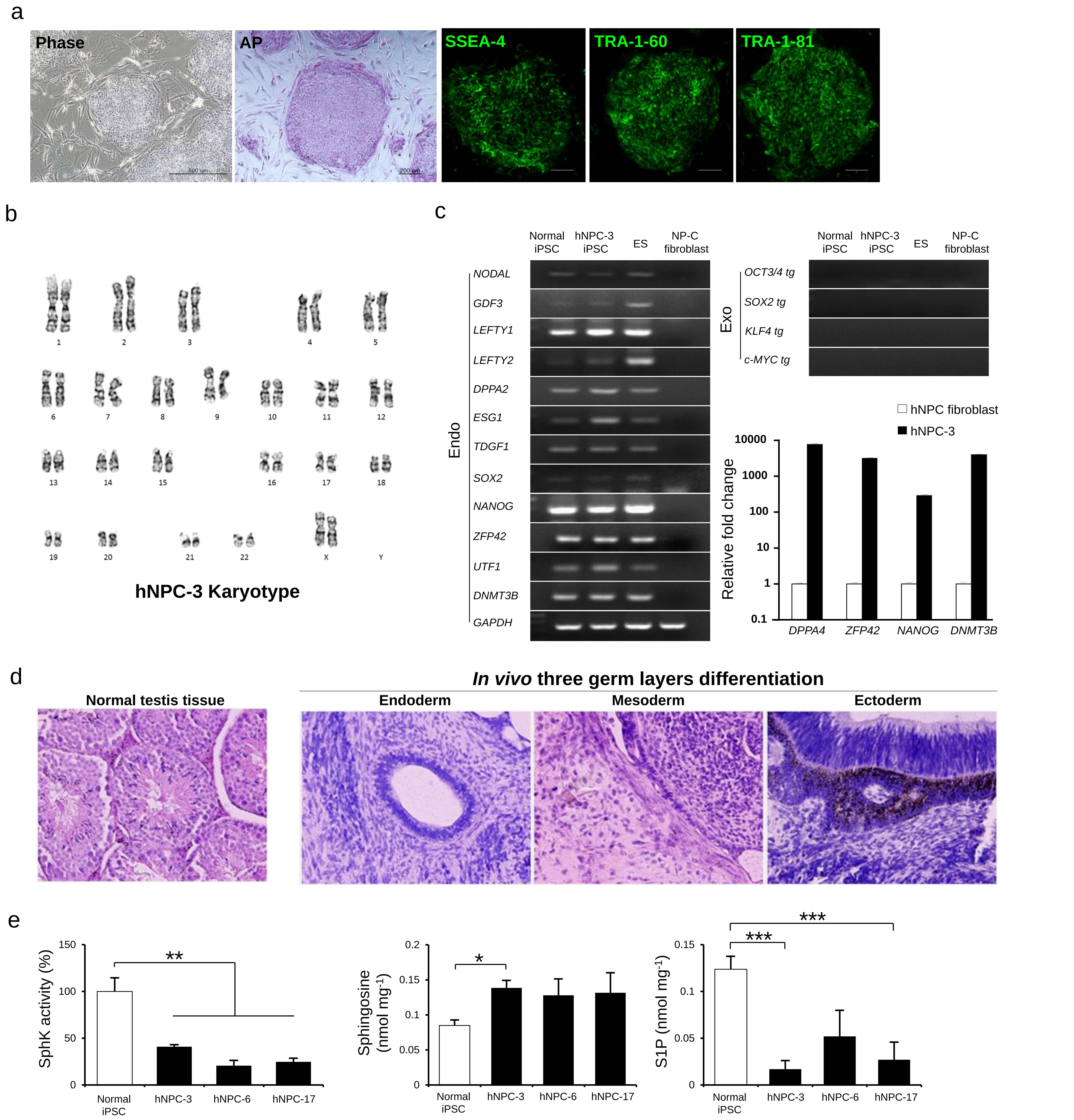

Supplementary Figure 8: Generation of NP-C iPSCs from patient fibroblasts. (**a**) Generation of NP-C iPSC lines from patient fibroblast. Established hNPC-3 iPSCs showed embryonic stem cell-like morphology (Phase) and expressed pluripotent stem cell markers alkaline phosphatase (AP), SSEA-4, TRA-1-60 and TRA-1-81 (scale bar in fluorescence images, 200 μm). (**b**) Normal karyotype of hNPC-3 iPSC line. (**c**) Reverse-transcription PCR showed reactivation of endogenous (endo) pluripotency genes and silencing of exogenous (exo) pluripotency genes in hNPC-3 line. Quantitative real-time PCR assays for *DPPA4*, *ZFP42*, *NANOG* and *DNMT3B* indicates increased expression in hNPC-3 iPSCs relative to parent cell line (hNPC fibroblast, $n = 6$; and hNPC-3 iPSCs, $n = 8$). (**d**) Representative series of hematoxylin-eosin stained sections from a formalin fixed teratoma produced from hNPC-3 cells (Magnification, X20). They formed mature, cystic teratomas with tissues representing all three embryonic germ layers including: respiratory epithelium (endoderm), bone and cartilage (mesoderm), and pigmented retinal epithelium (ectoderm). (**e**) SphK, sphingosine and S1P were estimated in normal and NP-C iPSC lines ($n = 7$ per group). e, one-way ANOVA, Tukey's post hoc test. $^{*}P < 0.05$, $^{**}P < 0.01$, $^{***}P < 0.005$. All error bars indicate s.e.m.

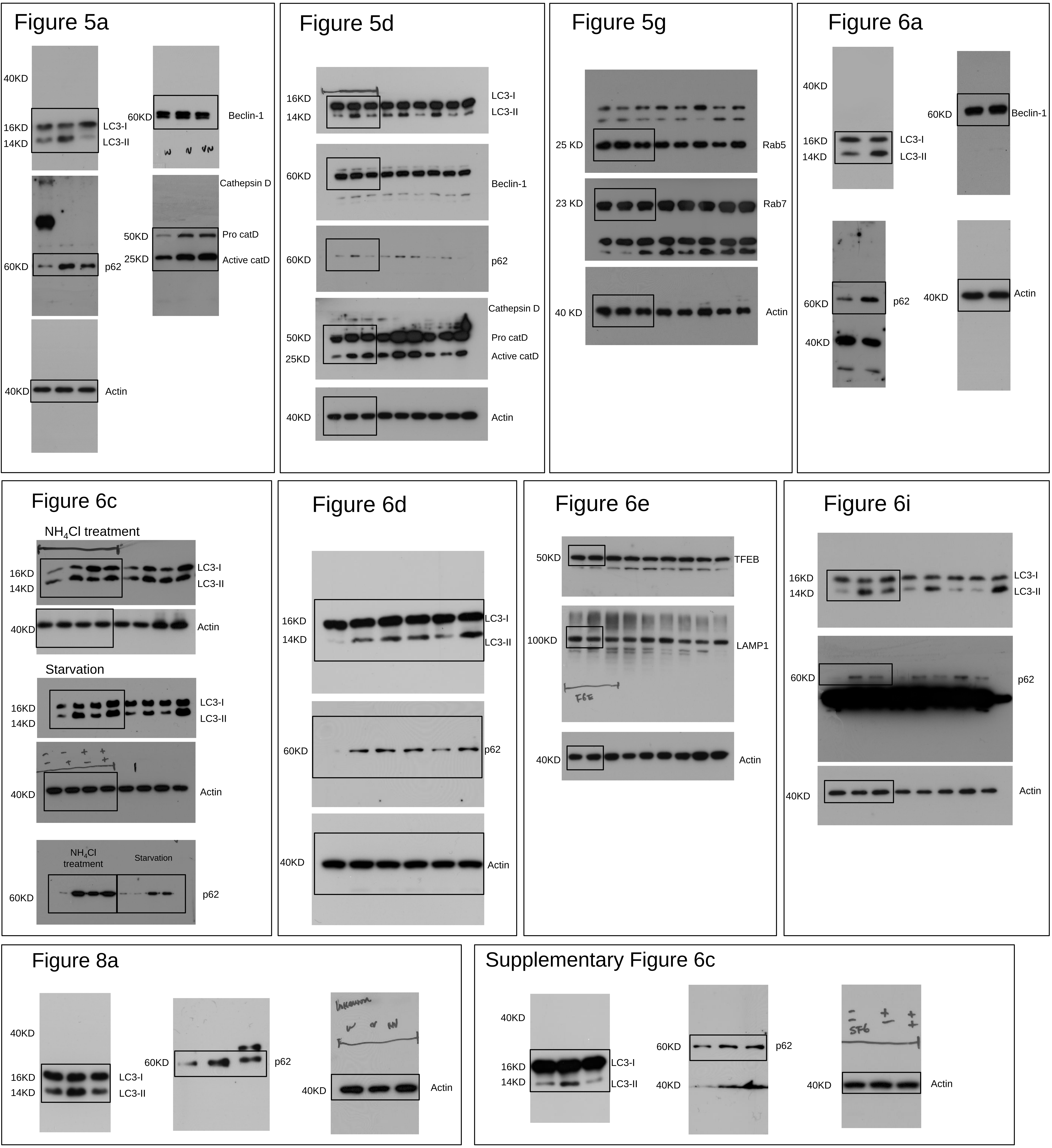

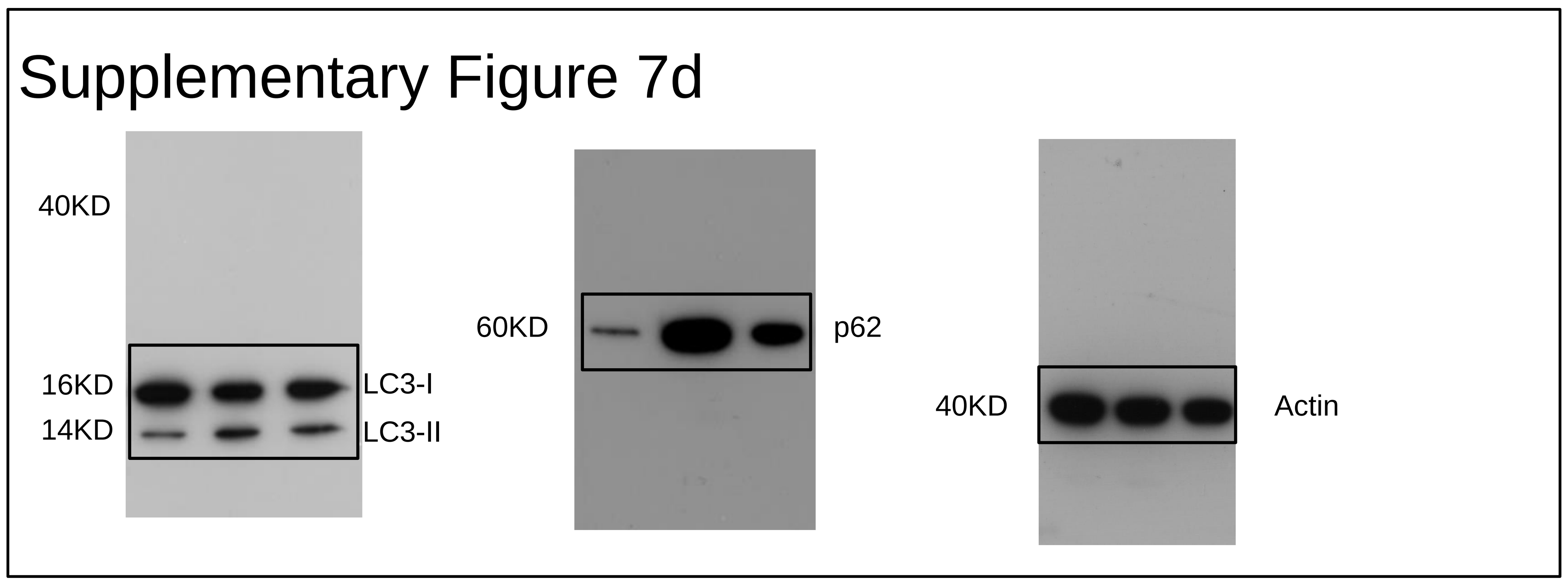

Supplementary Figure 9: Uncropped images of western blots included in figures.
